# Pathway-Consensus Approach to Metabolic Network Reconstruction for *Pseudomonas putida* KT2440 by Systematic Comparison of Published Models

**DOI:** 10.1371/journal.pone.0169437

**Published:** 2017-01-13

**Authors:** Qianqian Yuan, Teng Huang, Peishun Li, Tong Hao, Feiran Li, Hongwu Ma, Zhiwen Wang, Xueming Zhao, Tao Chen, Igor Goryanin

**Affiliations:** 1 Key Laboratory of Systems Bioengineering (Ministry of Education), School of Chemical Engineering and Technology, Tianjin University, Tianjin, P. R. China; 2 Key Laboratory of Systems Microbial Biotechnology, Tianjin Institute of Industrial Biotechnology, Chinese Academy of Sciences, Tianjin, China; 3 SynBio Research Platform, Collaborative Innovation Center of Chemical Science and Engineering (Tianjin), School of Chemical Engineering and Technology, Tianjin University, Tianjin, China; 4 College of Life Sciences, Tianjin Normal University, Tianjin, China; 5 School of Informatics, the University of Edinburgh, Informatics Forum, Edinburgh, United Kingdom; Universite Paris-Sud, FRANCE

## Abstract

Over 100 genome-scale metabolic networks (GSMNs) have been published in recent years and widely used for phenotype prediction and pathway design. However, GSMNs for a specific organism reconstructed by different research groups usually produce inconsistent simulation results, which makes it difficult to use the GSMNs for precise optimal pathway design. Therefore, it is necessary to compare and identify the discrepancies among networks and build a consensus metabolic network for an organism. Here we proposed a process for systematic comparison of metabolic networks at pathway level. We compared four published GSMNs of *Pseudomonas putida* KT2440 and identified the discrepancies leading to inconsistent pathway calculation results. The mistakes in the models were corrected based on information from literature so that all the calculated synthesis and uptake pathways were the same. Subsequently we built a pathway-consensus model and then further updated it with the latest genome annotation information to obtain modelPpuQY1140 for *P*. *putida* KT2440, which includes 1140 genes, 1171 reactions and 1104 metabolites. We found that even small errors in a GSMN could have great impacts on the calculated optimal pathways and thus may lead to incorrect pathway design strategies. Careful investigation of the calculated pathways during the metabolic network reconstruction process is essential for building proper GSMNs for pathway design.

## Introduction

Since the first GSMN was published in 1999 [1], more than 100 GSMNs have been reconstructed and an increasing number of GSMNs will be available with the development of semi-automated technologies for high-throughput generation of GSMNs [2–4]. GSMNs have been applied to the estimation of the growth rate, prediction of gene essentiality and computation of the optimal pathways from a specific substrate to a target metabolite [5–7]. However, GSMNs for a specific organism reconstructed by different research groups usually generate inconsistent simulation results. For example, the optimal growth rates from two yeast models (Yeast 5 and Yeast 4) were calculated to be 0.09 h^-1^ and 0.17 h^-1^ respectively with the same glucose input flux [8]. It is difficult to compare metabolic networks reconstructed by different groups directly as they often tend to use different compound names for the same metabolite without cross-links to standard chemical databases such as KEGG.

To address this problem, efforts have been made to create a consensus metabolic network by merging GSMNs for the same organism. The first consensus metabolic network, Yeast 1, was created from available yeast networks iMM904 and iLl672 by using a jamboree approach [9]. The jamboree approach provided the opportunity to establish common standards for the consensus reconstruction by using the same terminologies to describe the same chemical entities. With the successful achievement of the first consensus network, such a jamboree method has been used to build consensus reconstruction of *Salmonella Typhimurium* LT2 [10] and Human metabolic network Recon2 [11] which has been updated to Recon2.2 [12]. A drawback of such community driven reconstruction of consensus GSMN is that it is very time consuming and labor intensive, requiring many researchers (including those who reconstructed the original models) to work together to determine the standard terms and processes. The lack of strict quality control in the merging process could generate new problems in the network, sometimes even making the network infeasible for constraint-based analysis due to the insufficient pathway connectivity such as in Yeast 1.0 [13].

One of the important applications of GSMNs is to design optimal pathways for the production of biochemicals. Using methods like flux-balance analysis (FBA) [14,15], researchers can calculate pathways with the maximal yield for a specific product in a metabolic network and thus provide guidances for engineering the network to divert metabolic flows toward the optimal pathway. Considering this application, we propose a new approach to compare reconstructed GSMNs of a certain organism at pathway level and build a pathway-consensus metabolic network. With this method, it is possible to identify and correct the errors leading to discrepancies between different networks and subsequentely obtain consistent pathway analysis results. We applied this method to build a pathway consensus metabolic network for *Pseudomonas putida* KT2440 based on four previously published metabolic network models, iJN746 [16], iJP815 [17], PpuMBEL1071 [18] and iJP962 [19]. Actually iJP962 was reconstructed using a so called “metabolic network reconciliation” process which compared the networks of closely related organisms to eliminate errors in a network. The “network reconciliation” process is mainly based on genome level comparson and does not often include the systematic pathway comparison step. Whereas our “pathway-consensus” approach is focused on the pathway level comparison of different networks for the same organism and thus is more suitable for building a high quality network useful for reliable pathway design.

The original analysis results from the four *P*. *putida* models were quite different. iJP815 and iJP962 were reconstructed by the same research group and had smaller differences in the growth yield (92.4 gDCW·molGlc^-1^ *vs* 101.1 gDCW·molGlc^-1^) [19]. By comparing a series of calculated pathways from the four models, errors causing discrepancies were found out and then corrected based on information from literature and databases [20,21]. As the result of pathway comparison, a pathway consensus metabolic model was developed for *P*. *putida* KT2440 and further improved with updated genome annotation information to obtain model PpuQY1140 for *P*. *putida* KT2440. Compared with previous models, the simulation results from the new model are in better agreement with published experimental data.

## Results

### Workflow of the reconstruction process

**Fig 1:**
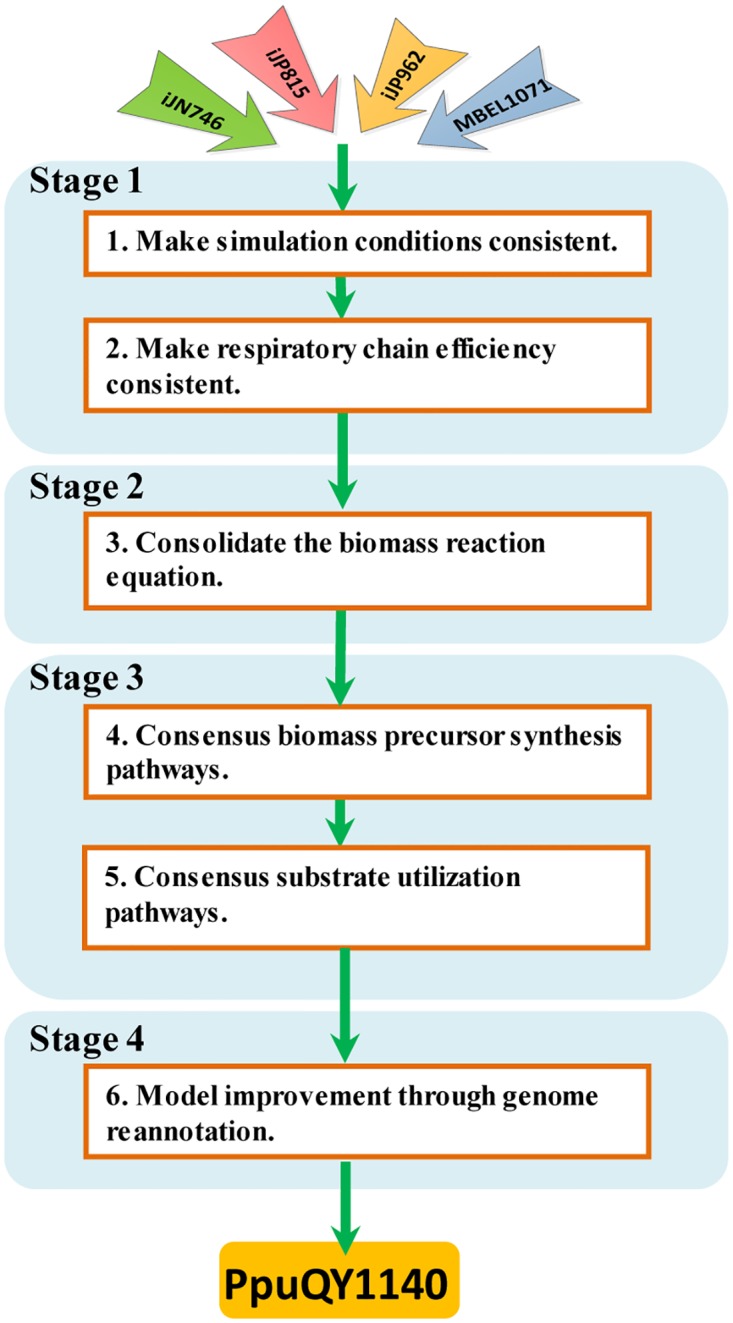
The workflow of the pathway-consensus metabolic network reconstruction of *Pseudomonas putida* KT2440.

The pathway-consensus metabolic network reconstruction was bulit by taking the systematic comparison of published *Pseudomonas putida* KT2440 models through a process summarized in Fig 1. The process includes: (1) processing the models to make simulated conditions and respiratory chain efficiency consistent in the four models; (2) consolidating the biomass reaction equation according to the measured biomass elemental composition and the mass balance constraints; (3) comparing the biosynthesis pathways and substrate utilization pathways from the models and obtaining consensus pathways by correcting errors; (4) model improvement through genome reannotation. The result of this process is a pathway-consensus model PpuQY1140for *P*. *putida* KT2440.

### Simulation results for the four GSMNs of *P*. *putida* KT2440

Four metabolic networks of *P*. *putida* KT2440, iJN746, iJP815, PpuMBEL1071 and iJP962, have been reconstructed by three research groups. The optimal growth rate can be calculated by performing FBA with the biomass production reaction as objective function. The optimal ATP production rate can be calculated by using the ATP maintenance reaction as objective function [14]. The simulated conditions containing exchange reaction boundaries and maintenance parameters (growth and non-growth related maintenance requirement for ATP) were set the same before calculation (see Materials and Methods for further details). The calculated optimal growth rates and ATP production rates of the four models were shown in Table 1.

**Table 1:** The optimal growth rates and ATP production rates calculated for the four published models of *P*. *putida* KT2440.

	iJN746	iJP815	PpuMBEL1071	iJP962
**Growth rate (h**^**-1**^**)**	0.909^a^	0.703^a^	0.460^a^	0.742^a^
	0.988^b^	0.783^b^	0.382^b^	0.748^b^
	0.850^c^	0.781^c^	0.783^c^	0.812^c^
	0.814^d^	0.814^d^	0.814^d^	0.814^d^
**ATP production rate (mmol·gDW**^**-1**^**·h**^**-1**^**)**	217.5^a^	192.5^a^	999999^a^	237.5^a^
	237.5^b^	237.5^b^	237.5^b^	237.5^b^

^a^ Results from the four original models. The maximal glucose uptake rates were set the same at 10 mmol·gDCW^-1^·h^-1^.

^b^ Using the same set of respiratory chain reactions.

^c^ Using the same biomass growth reaction equation.

^d^ Values calculated from the updated models where the precursor synthetic pathways were in consistence.

It can be seen that there was nearly a two-fold difference between the highest and lowest growth rates (0.909 h^-1^ compared to 0.46 h^-1^). More surprisingly, the obtained ATP production rate from PpuMBEL1071 was 999999 mmol·gDCW^-1^·h^-1^ which was actually the upper boundary of the reactions in that model. The ATP production rate remained constant at the value of the upper boundary even when we set glucose uptake rate to zero. This indicated that ATP was generated infinitely in PpuMBEL1071 without substrate consumption, which was obviously unreasonable for a biological network model. ATP plays an important role in cell metabolism as energy carrier. The generation and consumption of most metabolites are linked with energy metabolism, and thus mistakes in ATP production may also lead to incorrect calculation results for pathways to other metabolites. Therefore, we first analyzed the optimal ATP production pathways from the four models to identify the mistakes and correct them.

**Fig 2:**
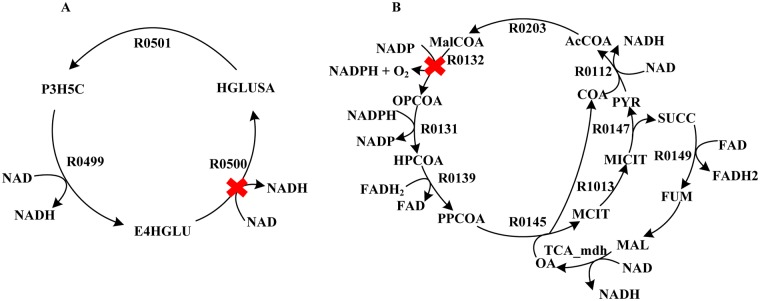
Incorrect reaction loops for unlimited NAD(P)H generation in PpuMBEL1071. (A) Incorrect reaction R0500 led to a loop to produce NADH without consuming any substrates. (B) Incorrect reaction R0132 led to a loop to produce NADPH and O_2_. P3H5C, L-1-Pyrroline-3-hydroxy-5-carboxylate; E4HGLU, L-erythro-4-Hydroxyglutamate; HGLUSA, L-4-Hydroxyglutamate semialdehyde; MALCOA, Malonyl-CoA; OPCOA, 3-Oxopropionyl-CoA; HPCOA, 3-Hydroxypropionyl-CoA; PPCOA, Propanoyl-CoA; MCIT, 2-Methylcitrate; MICIT, cis-2-Methylaconitate; PYR, Pyruvate; AcCOA, Acetyl-CoA; SUCC, Succinate; FUM, Fumarate; MAL, (S)-Malate; OA, Oxaloacetate.

From the calculated optimal ATP generation pathways in PpuMBEL1071 we found that there were two incorrect NAD(P)H generation loops as shown in Fig 2. NADH can be generated unlimitedly from these two loops without consuming any substrate and eventually produced unlimited ATP through respiratory chain reactions. By checking the corresponding reactions in KEGG [20] and MetaCyc [21] databases we found that the reaction equations for reactions R0500 (Eq 1) and R0132 (Eq 2) in the model were wrong. The NAD(P)/NAD(P)H pair were placed on the wrong sides of the reaction equations. These reactions should be corrected as Eqs 3 and 4, respectively.

$$\begin{array}{l} \text{L-erthro-}4\text{-hydroxyglutamate}+{\text{NAD}}^{+}↔\\ \text{L-}4\text{-hydroxyglutamate semialdehyde}+\text{NADH}+{\text{H}}^{+} \end{array}$$ (1)

$$3\text{-Oxopropionyl-CoA}+{\text{O}}_{2}+\text{NADPH}↔\text{Malonyl-CoA}+{\text{NADP}}^{+}$$ (2)

$$\begin{array}{l} \text{L-erthro-}4\text{-hydroxyglutamate}+\text{NADH}+{\text{H}}^{+}↔\\ \text{L-}4\text{-hydroxyglutamate semialdehyde}+{\text{NAD}}^{+}+{\text{H}}_{2}\text{O} \end{array}$$ (3)

$$3\text{-Oxopropionyl-CoA}+{\text{NADP}}^{+}+{\text{H}}_{2}\text{O}↔\text{Malonyl-CoA}+\text{NADPH}+{\text{H}}^{+}$$ (4)

After correcting the two reactions, there was no NAD(P)H production from the two loops and the optimal ATP production rate of PpuMBEL1071 was changed to a more reasonable value of 225 mmol·gDCW^-1^·h^-1^.

**Fig 3:**
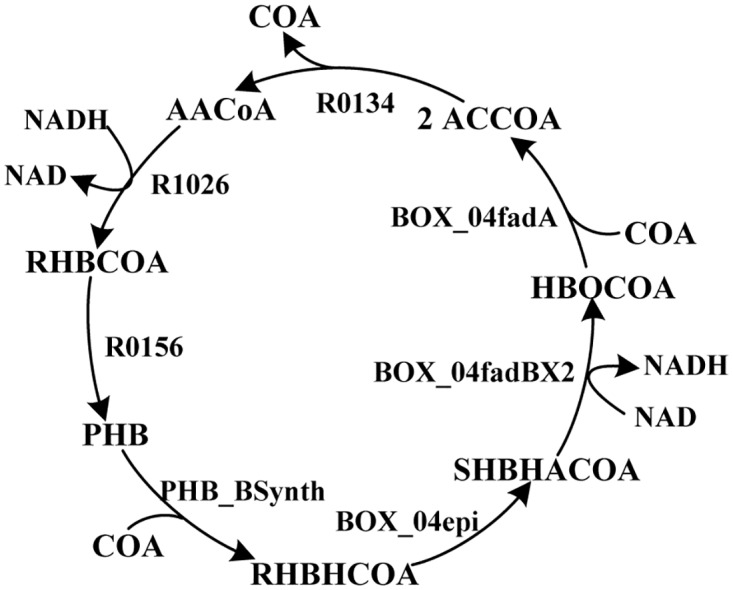
An unconserved reaction sets leading to a net consumption of CoA (defined as an unconserved metabolite) in PpuMBEL1071.

In view of the incorrect reaction loops caused by incorrect reaction equations, we further investigated if this kind of loops can be detected by using the method proposed by Gevorgyan et al [22] for checking stoichiometric inconsistencies in a network. By using their algorithm we detected 668 unconserved metabolites and the corresponding reaction sets causing the inconsistencies in PpuMBEL1071. For example, CoA was identified as an unconserved metabolite caused by the inconsistent reaction set shown in Fig 3. The overall reaction for this reaction set is “CoA→Ø”, a net consumption of CoA. The error was caused by the wrong reaction equation for R0156 where CoA should also be produced. Both the stoichiometric inconsistencies and the incorrect NAD(P)H generation loops were caused by the non-balanced reaction equations. However, we found that the incorrect reaction loops could not be discovered by the algorithms for checking stoichiometric inconsistencies. The overall reaction for a stoichiometric inconsistent reaction set is always the net production or consumption of a metabolite (defined as unconserved). Whereas the overall reaction for an incorrect reaction loop is the net conversion of NAD(P) to NAD(P)H. Both NAD(P) and NAD(P)H were not identified as unconserved. Therefore the incorrect reaction loops and the stoichiometric inconsistencies are two different kinds of errors in the network and both need to be corrected. We also investigated the stoichiometric inconsistencies in other models and found that only iJP962 did not contain such errors. Therefore we chose iJP962 as the reference to build a pathway-consensus model.

### Consistency of respiratory chain efficiency

**Fig 4:**
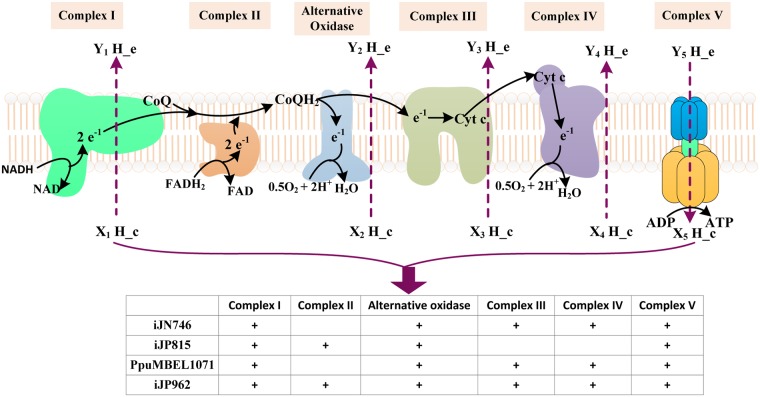
The respiratory chain reactions in the four models. ‘+’ shows that the complex exists in the model. The numbers of protons pumped from each complex (the value of X and Y) were summarized in Table 2.

Correcting the wrong reaction equations solved the problem of unlimited ATP production. However, the optimal ATP production rates from the four models were still different. ATP is mainly produced through the oxidative phosphorylation process in *P*. *putida* KT2440 [23]. Therefore we further investigated the electron transport chain reactions in the four models (Fig 4 and Table 2). Differences existed in the completeness of respiratory chain and the number of protons pumped out from an electron transfer reaction in the four models. For instance, the ferricytochrome mediated electron transfer reactions were missing in iJP815. Whereas in iJN746 and PpuMBEL1071, only NADH but not FADH_2_ could be oxidized through the respiratory chain (Fig 4).

**Table 2:** The respiratory chain reactions in the four models.

Complexes	Respiration reactions	iJN746	iJP815	1071	iJP962
**Complex I**	NADH+Q+X_1_H_c→NAD+Y_1_H_e+ QH_2_	X_1_ = 4,Y_1_ = 3 (X_1_ = 1, Y_1_ = 0)	X_1_ = 4.5, Y_1_ = 3.5 (X_1_ = 1, Y_1_ = 0)	X_1_ = 0, Y_1_ = 3 (X_1_ = 0, Y_1_ = 0)	X_1_ = 4.5, Y_1_ = 3.5
**Complex II**	FADH_2_+Q+XH_c→FAD+ QH_2_+YH_e	--	X = 0,Y = 0	--	X = 0,Y = 0
**Alternative Oxidase**	0.5O_2_+X2H_c+QH_2_→Q+Y_2_H_e+H_2_O	X_2_ = 4, Y_2_ = 4 (X_2_ = 2, Y_2_ = 2)	X_2_ = 2.5, Y_2_ = 2.5	X_2_ = 0, Y_2_ = 4 (X_2_ = 0, Y_2_ = 2)	X_2_ = 2.5, Y_2_ = 2.5 (X_2_ = 2, Y_2_ = 2)
**Complex III**	QH_2_+2Fic+X_3_H_c→Q+Y_3_H_e+2Foc	X_3_ = 2, Y_3_ = 2	--	X_3_ = 0, Y_3_ = 0	X_3_ = 0, Y_3_ = 2
**Complex IV**	0.5O_2_+X_4_H_c+2Foc→2Fic+Y_4_H_e+H_2_O	X_4_ = 0, Y_4_ = 0	--	X_4_ = 0, Y_4_ = 0	X_4_ = 4, Y_4_ = 2 (X_4_ = 3, Y_4_ = 1)
**Complex V**	PI+Y_5_H_e+ATP→ATP+X_5_H_c+H_2_O	X_5_ = 3, Y_5_ = 4	X_5_ = 3, Y_5_ = 4	X_5_ = 3, Y_5_ = 4	X_5_ = 3, Y_5_ = 4

Q: Ubiquinone-8, QH_2_: Ubiquinol-8, Fic: Ferricytochrome c, Foc: Ferrocytochrome c

‘--’: the complex and reaction did not appear in the model

‘()’: if there are more than one respiration chain reactions for the same complex, the one with smaller coefficients is placed in parenthesis.

In order to clearly recognize the efficiency of the respiratory chains, we combined a series of respiratory chain reactions into one overall reaction for each model to get the P/O ratio. It can be seen from Table 3 that the ATP production efficiencies (represented by P/O ratios) are quite different in the four models and this may explain the differences in the optimal ATP production rates. The respiratory chains from iJP962 are the most complete with the highest efficiency and agree well with the actual respiratory chain in *P*. *putida* KT2440 [23]. By replacing the respiratory chain reactions in other models by those in iJP962 we obtained the same optimal ATP production rates of 237.5 mmol·gDCW^-1^·h^-1^ for the four models.

**Table 3:** The overall respiratory chain reactions of the four models.

Model	Combined overall reaction	P/O ratio
**iJN746**	4NADH+11H+2O_2_+7ADP+7PI→7ATP+4NAD+11H_2_O	1.75
**iJP815**	2NADH+5H+O_2_+3ADP+3PI→3ATP+2NAD+5H_2_O	1.5
	8FADH_2_+5H+4O_2_+5ADP+5PI→5ATP+8FAD+13H_2_O	0.625
**iJP962**	8NADH+23H+4O_2_+15ADP+15PI→15ATP+8NAD+23H_2_O	1.85
	FADH_2_+H+0.5O_2_+ADP+PI→ATP+FAD+H_2_O	1.0
**PpuMBEL1071**	4NADH+2O_2_+7ADP+7PI→7ATP+4NAD	1.75

### Consolidate the biomass reaction equation

After revising the models to obtain consistent ATP production rates, we further calculated the optimal growth rates of the four models (values marked with ‘^b^’ in Table 1) and found that they were still quite different. To find the reasons, we investigated the biomass production equations in the four models (S1 Table). The biomass reaction can normally be written in a format as Eq 5

$$\underset{\text{i}}{\sum }{\text{S}}_{\text{i}}^{\text{R}}{\text{R}}_{\text{i}}=\text{Biomass}+\underset{\text{j}}{\sum }{\text{S}}_{\text{j}}^{\text{B}}{\text{B}}_{\text{j}}$$ (5)

Where ${\text{S}}_{\text{i}}^{\text{R}}$ is the stoichiometric coefficient of metabolite R_i_, that is, the growth requirement of metabolite R_i_ (often in mmol) for the production of 1 gram dry cell weight. ${\text{S}}_{\text{i}}^{\text{B}}$ is the stoichiometric coefficient of by-product B_j_.

As all coefficients are determined based on 1 gram dry cell weight, the biomass reaction should satisfy the mass balance constraint that can be written as Eq 6

$$(\underset{\text{i}}{\sum }{\text{S}}_{\text{i}}^{\text{R}}{\text{M}}_{\text{i}}^{\text{R}}-\underset{\text{j}}{\sum }{\text{S}}_{\text{j}}^{\text{B}}{\text{M}}_{\text{j}}^{\text{B}})/1000=1\;\left(\text{g DCW}\right)$$ (6)

Where ${\text{M}}_{\text{i}}^{\text{R}}$, ${\text{M}}_{\text{j}}^{\text{B}}$ are the molecular mass of R_i_ and B_j_, respectively. However, none of the growth reactions in the four models satisfied this constraint (S1 Table). Especially for PpuMBEL1071, the coefficient values in the biomass production reaction are so high that 2.78 g instead of 1 g dry cell weight should be produced to satisfy mass balance. This may explain why the optimal growth rate of PpuMBEL1071 was far less than those of the other three models (Table 1).

In addition to the mass balance constraint, the coefficients should also satisfy the elemental balance, namely to match the experimentally measured C, H, O, N, P contents in the biomass. According to the experiment conducted by Passman and Jones, the biomass element composition of *P*. *putida* was determined to be CH_1.69_O_0.344_N_0.235_P_0.0133_ [24]. Therefore the elemental balance equations can be mathematically depicted as Eqs 7–10.

$$(\underset{\text{i}}{\sum }{\text{S}}_{\text{i}}^{\text{R}}{\text{H}}_{\text{i}}^{\text{R}}-\underset{\text{j}}{\sum }{\text{S}}_{\text{j}}^{\text{B}}{\text{H}}_{\text{j}}^{\text{B}})/\text{A}=1.69$$ (7)

$$(\underset{\text{i}}{\sum }{\text{S}}_{\text{i}}^{\text{R}}{\text{O}}_{\text{i}}^{\text{R}}-\underset{\text{j}}{\sum }{\text{S}}_{\text{j}}^{\text{B}}{\text{O}}_{\text{j}}^{\text{B}})/\text{A}=0.344$$ (8)

$$(\underset{\text{i}}{\sum }{\text{S}}_{\text{i}}^{\text{R}}{\text{N}}_{\text{i}}^{\text{R}}-\underset{\text{j}}{\sum }{\text{S}}_{\text{j}}^{\text{B}}{\text{N}}_{\text{j}}^{\text{B}})/\text{A}=0.235$$ (9)

$$(\underset{\text{i}}{\sum }{\text{S}}_{\text{i}}^{\text{R}}{\text{P}}_{\text{i}}^{\text{R}}-\underset{\text{j}}{\sum }{\text{S}}_{\text{j}}^{\text{B}}{\text{P}}_{\text{j}}^{\text{B}})/\text{A}=0.0133$$ (10)

$$\text{A}=\underset{\text{i}}{\sum }{\text{S}}_{\text{i}}^{\text{R}}{\text{C}}_{\text{i}}^{\text{R}}-\underset{\text{j}}{\sum }{\text{S}}_{\text{j}}^{\text{B}}{\text{C}}_{\text{j}}^{\text{B}}$$

where *${\text{C}}_{\text{i}}^{\text{R}}$* and ${\text{C}}_{\text{j}}^{\text{B}}$ (or ${\text{H}}_{\text{i}}^{\text{R}}$ and ${\text{H}}_{\text{j}}^{\text{B}}$, ${\text{O}}_{\text{i}}^{\text{R}}$ and ${\text{O}}_{\text{j}}^{\text{B}}$, ${\text{N}}_{\text{i}}^{\text{R}}$ and ${\text{N}}_{\text{j}}^{\text{B}}$, ${\text{P}}_{\text{i}}^{\text{R}}$ and ${\text{P}}_{\text{j}}^{\text{B}}$) are the number of C (or H, O, N, P) atoms in R_i_, B_j_. However, the biomass reactions of the four models were not in conformity with the elemental balance. To address this problem, we manually adjusted the precursors’ coefficients mainly based on the values in the biomass reaction equation of iJP962 to satisfy the mass balance and elemental balance (Eqs 6–10) and obtained a new biomass reaction (S2 Table) to replace those in the original models. The missing synthesis pathways of precursors and corresponding genes which existed in iJP962 but not in the others were also added to the corresponding models. For example, the glycogen synthetic pathway and related genes which were missing in iJN746 were added into the model. However, even after using the same biomass reaction the calculated optimal growth rates from the four models were still different (values marked ‘^c^’ in Table 1), implying other inconsistencies in the synthesis pathways for the biomass building blocks.

### Compare pathways for biomass precursors production

We calculated the optimal production rates for all 47 biomass precursors by adding a demand reaction [25,26] for each biomass precursor and found that many of them were different in the four models (S3 Table, results for 20 amino acids were also shown in Table 4). Actually only for 2 precursors, L-valine and L-alanine, the optimal rates were the same in all four models. We carefully examined the optimal precursor synthesis pathways one by one in the four models. This cross examination enables us to find the causes for the differences which were described in detail below.

**Table 4:** The calculated optimal rates of 20 amino acids from the models.

	iJN746	iJP815	PpuMBEL1071	iJP962	Pathway-consensus model
**L-Valine**	10	10	10	10	10
**L-Asparagine**	17.39	15	13.33	15	15.12
**L-Tryptophan**	4.612	4.278	4.289	4.278	4.279
**L-Alanine**	20	20	20	20	20
**L-Methionine**	10	6.316	6.764	6.493	6.679
**Glycine**	40	30	31.62	30	28.24
**L-Histidine**	9.902	8.297	7.5	8.297	8.297
**L-Arginine**	9.739	8.986	8	8.342	8.986
**L-Lysine**	8.404	7.882	7.735	7.882	7.882
**L-Phenylalanine**	5.295	5.279	5.506	5.279	5.283
**L-Cysteine**	13.33	11.07	10.55	11.07	10.55
**L-Proline**	10.15	10.09	10	10.09	10.10
**L-Aspartate**	18.79	15	13.33	15	18.79
**L-Glutamine**	11.98	11.06	10	11.06	11.18
**L-Serine**	24	20	20	20	20
**L-Leucine**	8	7.398	6.667	7.398	7.482
**D-Glutamate**	12.58	11.35	10	11.35	11.51
**L-Tyrosine**	5.523	5.504	5.506	5.504	5.506
**L-Threonine**	15	12.83	13.33	12.83	12.83
**L-Isoleucine**	8	7.411	7.767	7.411	7.411

#### 1. Errors in reaction direction and reaction reversibility

**Fig 5:**
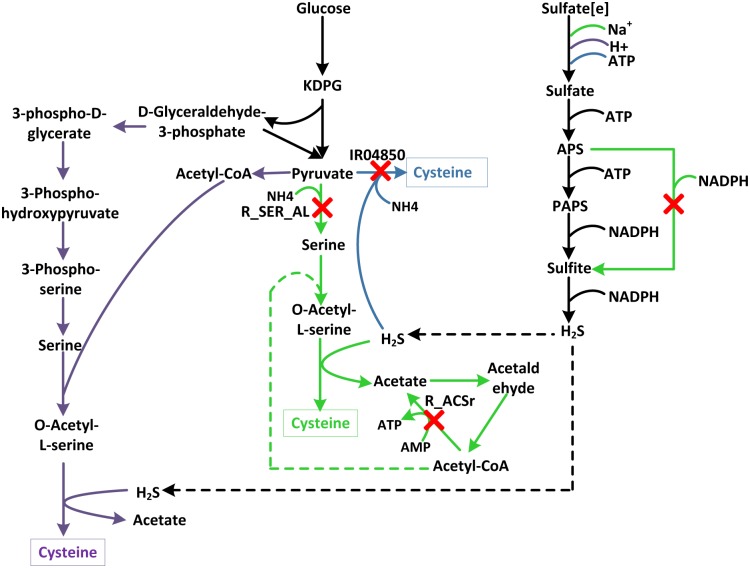
Cysteine synthesis pathway in the four models. Only the pathway in PpuMBEL1071 was right. Green, blue and purple arrows depict reactions in iJN746, iJP815 (iJP962) and PpuMBEL1071 respectively. Wrong directions of R_SER_AL and R_ACSr (in iJN746), IR04850 (in iJP815 and iJP962) led to wrong cysteine synthesis pathways. KDPG: 2-Dehydro-3-deoxy-6-phospho-D-gluconate.

For example, the optimal cysteine production rates in iJN746, iJP815, PpuMBEL1071 and iJP962 is 13.33, 11.07, 10.547 and 11.07 mmol·gDCW^-1^·h^-1^, respectively and the optimal pathways were shown in Fig 5. Only the pathway in PpuMBEL1071 was the same with the widely recognized cysteine synthesis pathway in bacteria where cysteine is synthesized from serine through acetylation and sulfide incorporation [27]. The pathways in iJP815 and iJP962 were the same where cysteine was produced directly from pyruvate by the reaction IR04850 (Eq 11). However, reaction IR04850 should be irreversible and can only be carried out on the direction toward desulphonation [28]. A similar problem existed for R_SER_AL (Eq 12) in iJN746 where serine was produced directly from pyruvate. Actually serine is mainly synthesized from 3-phosphoglycerate in bacteria [29] and R_SER_AL can only be carried out toward pyruvate production under physiological conditions. Another reversibility problem in reaction R_ACSr (Eq 13) in iJN746 led to a cycle for ATP net generation without consuming any substrates. This thermodynamically infeasible pathway may explain the higher production rates of cysteine and many other precursors calculated for iJN746.

$$\text{Pyruvate}+\text{Hydrogen sulfide}+{\text{NH}}_{4}\to \text{Cysteine}+{\text{H}}_{\text{2}}\text{O}$$ (11)

$$\text{Serine}↔{\text{H}}^{+}+{\text{NH}}_{3}+\text{Pyruvate}$$ (12)

Acetate+ATP+CoA↔Acetyl-CoA+AMP+PPi (13)

#### 2. Missing reactions

In above-mentioned example, the energy consumption for sulfate transport and metabolism was different in the four models (Fig 5). Two sulfate-activating enzymes (ATP-sulfurylase and APS kinase) exist in *P*. *putida* to catalyze the activation of inorganic sulfate to APS (Adenylyl sulfate) and then to PAPS (adenosine-5-phosphosulfate) [30]. PAPS is converted into sulfite with NADPH consumption. In iJN746, APS could be converted to sulfite directly without PAPS generation and ATP consumption. Therefore the production rates of sulfur-containing metabolites such as L-cysteine and L-methionine in iJN746 were higher than the other three models. The reaction from APS to PAPS and corresponding genes should be added into the model.

#### 3. Errors in reaction equations

As described in the ATP production pathway analysis section, incorrect reaction equation led to unlimited ATP production. Here by investigating the precursor synthesis pathways, we found more mistakes in reaction equations in all four models. For example in PpuMBEL1071 the reaction equation of R0749 was Folate + NADPH → Tetrahydrofolate + NADP^+^. The numbers of reactions corrected for all the three types of errors in the four models were shown in Table 5 and the detailed information of model correction were shown in S1 File. With model revision based on pathway level comparison, the optimal production rates for all 47 biomass precursors were the same in the four models (Table 4 shows the consensus values for 20 amino acids and the complete list can be seen in S3 Table) and the optimal growth rates were also exactly the same as 0.814 h^-1^. The calculated sum of squared errors indicated that the results from iJP815 and iJP962 were close to those from the consensus network while iJN746 and PpuMBEL1071 had more errors in the model.

**Table 5:** Summary of the number of errors corrected for the pathway-consensus process^a^.

	iJN746	iJP815	PpuMBEL1071	iJP962
**Errors in reaction direction**	12 (1)	11 (2)	8 (3)	7 (2)
**Errors in reaction equation**	6 (3)	7 (0)	15 (0)	5 (0)
**Missing reactions**	17 (6)	20 (20)	30 (18)	16 (20)

^a^: the numbers shown in parentheses indicate the number of errors corrected in the substrate utilization pathway consensus step.

### Analysis of substrate utilization pathways

**Fig 6:**
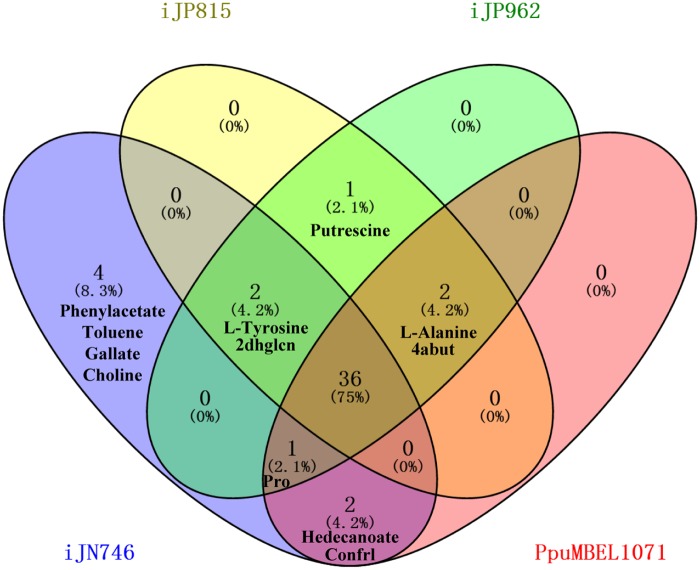
Utilization of 48 substrates in the four models for *P*. *putida* KT2440. 36 substrates could be utilized in the four original models. Phenylacetate, toluene, gallate and choline could only be utilized in iJN746. Putrescine could be utilized in iJP815 and iJP962. 2dhglcn: 2-Dehydro-D-gluconate, 4abut: 4-Aminobutanoate, Confrl:Conifery-alcohol.

In all the above analysis glucose was used as the sole carbon source. Whereas *P*. *putida* KT2440 is well known for its ability to grow on many amino acids and organics acids as carbon source [31,32]. Correspondingly in the existing metabolic network models a large number of reactions were dedicated for the utilization of various substrates. Therefore it is also important to compare the calculated pathways for substrate utilization in the four models. We compared the calculated aerobic growth capabilities of the four models using 48 different carbon sources known to be used by *P*. *putida* [17,31,33–36] (S4 Table). We found that 11 out of the 48 substrates could not be utilized in at least one model (Fig 6). For these substrates, we took the uptake pathways in other models as references to add new reactions and thus enable their utilization in the corresponding models. For example, as already mentioned in the original publication, alanine could not be used as a carbon source in iJN746 [16]. By comparing with the alanine uptake pathways in other models we found that this was due to the lack of transport reaction (L-alanine_p ↔ L-alanine_c) which transfer alanine from periplasm to cytoplasm. After adding this reaction, alanine could be used as a carbon source in iJN746. In a similar way we added 30 reactions and their corresponding genes to the four models (S2 File) and finally all 48 substrates could be used in four models.

However, there are still quantitative differences on the optimal growth rates calculated from different models. Actually we found that for 36 substrates the growth rate calculated with PpuMBEL1071 was higher than those obtained with other models. By investigating the pathways we revealed that the main reason was the incorrect reversibilty information of the reaction ED_edd (Eq 14) catalyzed by phosphogluconate dehydratase and reaction ED_eda (Eq 15) catalyzed by 2-dehydro-3-deoxy-phosphogluconate aldolase.

$$\begin{array}{l} 6\text{-Phospho-D-gluconate}↔\text{KDPG}+{\text{H}}_{2}\text{O}\\ \left(\text{KDPG}:\;2\text{-Dehydro-}3\text{-deoxy-D-gluconate-}6\text{-phosphate}\right) \end{array}$$ (14)

KDPG↔D-glyceraldehyde 3-phosphate+Pyruvate (15)

The two reactions were mistakenly set to be reversible and thus enabled the formation of D-Ribulose 5-phosphate by the opposite direction of Entner-Doudoroff (ED) pathway (G3P + Pyr → 6-Phospho-D-gluconate → D-ribulose 5-phosphate) which consumed less energy for some substrates rather than pentose phosphate (PP) pathway. We corrected this error and recalculated the growth rates in PpuMBEL1071 for further comparison. The process was repeated untill the growth rates from all 48 substrates were the same in all four models. The number of added and modified reactions during this process were also shown in Table 5. The detailed information about corrected reactions for the four models can be seen in S2 File.

Four updated models of iJN746, iJP815, PpuMBEL1071 and iJP962 with more reactions and genes were obtained (S3–S6 Files). The basic information about the four original and updated pathway-consensus models were shown in Table 6. The updated version of iJP962was chosen for further improvement because of the following reasons: (1) it had the highest number of genes; (2) iJP962 was the original model with the best quality compared with the other three models.

**Table 6:** Summary statistics of the updated pathway-consensus models and the original models (in parentheses).

	iJN746	iJP815	PpuMBEL1071	iJP962
**Genes**	772 (746)	859 (815)	924 (900)	985 (962)
**Reactions**	1005 (950)	927 (877)	1117 (1071)	993 (973)
**Metabolites**	956 (911)	918 (886)	1062 (1044)	1008 (990)

### Model improvement through genome reannotation

The updated iJP962 model contains more genes than the original model but there are still around 80 metabolic genes existing in other models but not in the new model. Furthermore, many new enzyme genes have been found since the publication of the genome of *P*. *putida* KT2440 in 2002 [37]. To make the network more complete, we further extended the updated network by integrating the latest genome annotation information. We downloaded the genome of *P*. *putida* KT2440 from NCBI and submitted it to the RAST annotation server [38] for reannotation. We then carefully investigated the newly annotated enzyme genes (with an EC number) and found the proper reactions from databases like KEGG and MetaCyc to be added to the network (S7 File). We also compared the new reactions with the existing reactions in the model to avoid repeat reaction with different metabolite names. For example, PP_1806 was a new gene annotated as arabinose 5-phosphate isomerase. This enzymatic function was also encoded by gene PP_0957 which was already in the network. In this case we just added a new gene-reaction relationship between PP_1806 and the arabinose 5-phosphate isomerase catalyzed reaction RR03692 rather than added a new reaction. The reaction equations of the new reactions from KEGG/MetaCyc might also need to be changed so that the metabolite names are in consistent with those already in the network. Otherwise the new reactions could be disconnected from the main network. We also found some wrong gene-reaction relationships in the model during the re-annotation process. For example, the pyruvate carboxylation reaction RR00181 (ATP+Pyruvate+HCO_3_^-1^→H+ + Pi + ADP + Oxaloacetate) was linked with an nonexist gene PP_5437 in the updated iJP962 model. From the annotation information we found the right gene should be PP_5347 annotated as pyruvate carboxyl transferase subunit A (EC 6.4.1.1). Altogether we added 155 genes and 74 reactions. The final pathway-consensus metabolic model PpuQY1140 contains 1140 genes, 1171 reactions and 1104 metabolites (Table 7). PpuQY1140 in SBML file format is available in S8 File.

**Table 7:** Summary of PpuQY1140 model.

Total reactions (Gene associated)	1171 (979)
Biochemical reactions	942
Transport reactions	118
Exchange reactions	111
Metabolites	1104

## Model validation

**Fig 7:**
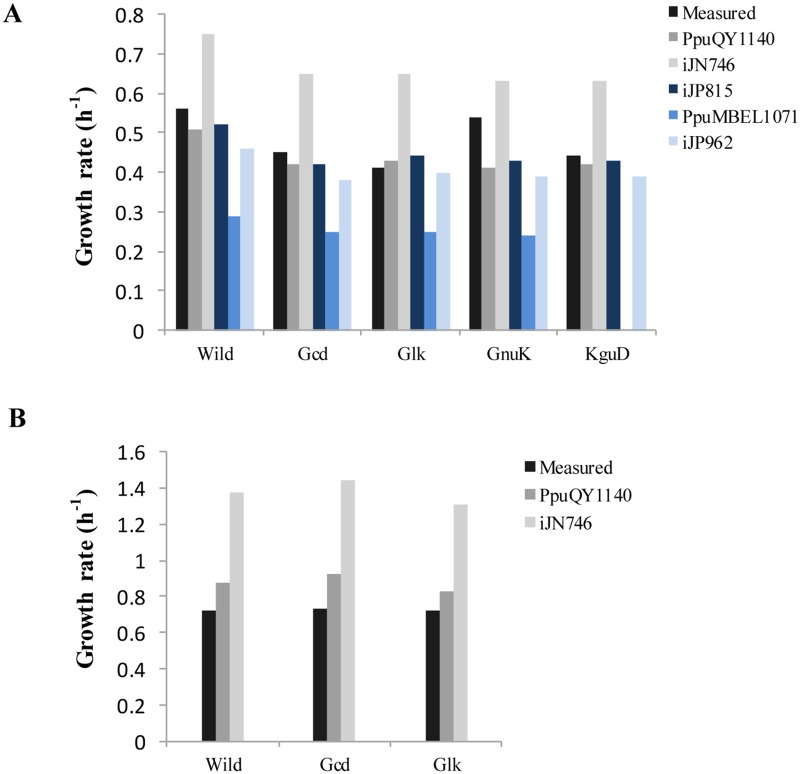
Experimentally measured and in silico predicted growth rates of *P*. *putida* KT2440 and the mutant strains. (A) Growth rates on glucose M9 medium. (B) Growth rates on toluene M9 medium.

To test the accuracy of the updated model PpuQY1140, we compared the simulated growth rates from different models with the experimentally measured values reported by del Castillo T et al [39]. They measured growth rates on M9 minimal medium with glucose of the wild type strain and four mutant stains (deletion of four genes *gcd*, *glk*, *gnuK*, *kguD* in 6-phosphogluconate synthesis pathway). The results shown in Fig 7A (detailed values in S5 Table) indicated that the simulated growth rates from the consensus model were very close to the experimental results except for the *gnuK* deletion strain which grew even faster than the wild type strain. This phenomenon might be caused by complex regulatory mechanisms and thus can not be explained by metabolic network analysis alone. Generally the simulation results from the consensus model are also better than those from other models, especially iJN746 and PpuMBEL1071. This is in agreement with the biomass precusor synthesis pathway simulation results as shown in Table 4. Actually the simulated growth rates from the consensus model are quite similar to those from iJN815 even though the calculated optimal pathways for many metabolites are different (e.g. 9 of 20 amino acids have different optimal synthesis rates in Table 4). This reflects the fact that accurate prediction of growth rates can not gurantee a correct metabolic model. Careful investigation at pathway level are necessary for high quality network reconstruction, especially when the model will be used for the design of optimal pathways for the production of biochemicals. In addition to growth characteristics of *P*. *putida* KT2440 and its deficient mutants growing on glucose minimal medium, the growth rates of wild type *P*. *putida* KT2440 and two deficient mutants (*gcd* and *glk*) growing on toluene M9 minimal medium were also tested by del Castillo T et al [40]. Only PpuQY1028 and iJN746 can use toluene as a single carbon source for growth (S4 Table) and thus we only calculated the growth rates of the two deficient mutants for these two models (Fig 7B and S5 Table). The results shown in Fig 7B indicated that the simulated growth rates of PpuMBEL1071 were in better agreement with the experimental results than those of iJN746.

## Discussion

We proposed a novel pathway-consensus process for identifying and solving the discrepancies between metabolic network reconstructions for the same organism by pathway level comparison. By applying this approach to four GSMNs of *P*. *putida* KT2440, all synthesis pathways for biomass building blocks and substrate uptake pathways were made consensus. Comparative analysis of the models revealed that small errors in reaction equations and reaction reversibility could greatly affect the pathway calculation results, sometimes even led to infeasible pathways such as the unlimited NADH generation loops. Therefore the pathway examination step should be included in the metabolic network reconstruction process to make sure that the quality of network is high enough for reliable pathway design.

This pathway-consensus approach should be regarded as complementary to the network reconciliation approach proposed by Oberhardt et al [19]. The network reconciliation approach mainly use the networks of closely related organisms as references for the reconstruction and consolidation of new networks. It improves the network quality by cross-organism comparison but the comparison is mainly at gene/reaction level rather than at pathway level. Therefore even though the network quality was improved by the network reconciliation process (e.g. iJP962), the calculated pathways from it may still be wrong as described in the paper (Table 4, Fig 5 etc). The pathway-consensus approach adds another level of quality improvement to the network reconciliation process to make sure all the calculated pathways are biologically reasonable and thermodynamically feasible. The pathway-consensus approach is time-consuming due to the requirement of manual comparison and examination of many calculated pathways. Whereas the network reconciliation can somehow be automated (e.g. transfer the reaction information from one organism to another based on sequence similarity). Therefore, for high quality network reconstruction, one may first use the reconciliation approach to add more reactions and then apply the pathway-consensus approach for reliable pathway design. It should be noted that though the pathway-consensus approach is mainly used for comparing networks of the same organism, it can also be extended for comparing networks of different organisms for pathway level consolidation and uncovering the true pathway differences between networks.

## Materials and Methods

### Model preprocessing

Four *P*. *putida* GSMNs in SBML format were obtained from supplementary materials of the publications [16–19]. The exchange reactions that used to define in silico medium conditions and their boundary values were shown in S6 Table. Here the growth was simulated in M9 minimal medium with glucose as the carbon source and the maximal glucose uptake rates was set at 10 mmol·gDCW^-1^·h^-1^. The uptake rates of nitrogen source, oxygen, phosphate and sulfur were not constrained. Exchange reactions were added to PpuMBEL1071 because there were no reactions for the utilization of different substrates and nutritions in this model. The growth-associated and non-growth-associated energy maintenance (GAM) and (NGAM) [41] parameters will affect the calculated optimal growth rate and they were different in four models (S7 Table). We preset them to the same values as those in iJP962.

### Flux balance analysis

Flux-balance analysis (FBA) [13] is a widely used approach for analysis of genome-scale metabolic networks. The COBRA Toolbox 2.0 [42], a MATLAB package, was used for FBA analysis. Linear optimizations (LP) were performed utilizing the free CPLEX (IBM, Armonk, NY, USA) solver. Loopless-FBA option was selected to obtain optimal metabolic pathways without loops that have no net consumption or production of any metabolite.

### Overall respiratory chain reaction and P/O ratio

The P/O ration represents the number of ATP molecules (P) formed from per oxygen atom (O) [27]. Here the P/O was obtained by combining a series of respiratory chain reactions into one overall reaction for each model. In this process, the most efficient respiratory chain was chosen, namely, the reactions in Table 2 which can produce more H_e were used to get the overall reaction. For example, in iJP962, the respiratory chain Eqs 16–19 was the most efficient respiratory chain for NADH oxidation.

$$\text{NADH}+\text{Q}+4.5\text{H}\_\text{c }\to \text{NAD}+3.5\text{H}\_\text{e}+{\text{QH}}_{2}$$ (16)

$${\text{QH}}_{2}+2\text{Fic}\to \text{Q}+2\text{H}\_\text{e}+2\text{Foc}$$ (17)

$$0.5{\text{O}}_{2}+4\text{H}\_\text{c}+2\text{Foc}\to 2\text{Fic}+2\text{H}\_\text{e}+{\text{H}}_{2}\text{O}$$ (18)

$$\text{PI}+4\text{H}\_\text{e}+\text{ADP}\to \text{ATP}+3\text{H}\_\text{c}+{\text{H}}_{\text{2}}\text{O}$$ (19)

The four reactions were combined to get the overall reaction (Eq 20) by eliminating the intermediates. From this overall reaction we can see that the P/O is 15/8 (15 ATP produced from 8 oxygen atoms).

$$8\text{NADH }+4{\text{O}}_{\text{2}}+23\text{H}\_\text{c}+15\text{ADP}+15\text{PI}\to 8\text{NAD}+15\text{ATP}+23\;{\text{H}}_{\text{2}}\text{O}$$ (20)

## Supporting Information

S1 Table Biomass compositions and precursor coefficients in four original models. (XLSX)

S2 Table The new biomass composition of *P*. *putida*. (XLSX)

S3 Table Optimal rates of biomass precursors when simulation conditions and efficiency of respiratory chain were set the same values. (XLSX)

S4 Table The calculated aerobic growth capabilities of four models using 48 different carbon sources. (XLSX)

S5 Table Comparison of simulated growth rates of four models with the experimentally measured values. (XLSX)

S6 Table The exchange reactions used to define *in silico* medium conditions in this work. (XLSX)

S7 Table The values of GAM and NGAM of four original models. (XLSX)

S1 File The detailed information about the reactions corrected in the pathway-consensus process with glucose as the carbon source. (DOCX)

S2 File The detailed information about reactions corrected when 48 substrates were used as individual carbon sources. (DOCX)

S3 File SBML format file for the updated version of iJN746. (XML)

S4 File SBML format file for the updated version of iJP815. (XML)

S5 File SBML format file for the updated version of PpuMBEL1071. (XML)

S6 File SBML format file for the updated version of iJP962. (XML)

S7 File Genes and reactions obtained from genome annotation analysis. (XLSX)

S8 File SBML format file for the final pathway-consensus metabolic network PpuQY1140. (XML)
