# Supplementary material for: Pathway-Consensus Approach to Metabolic Network Reconstruction for Pseudomonas putida KT2440 by Systematic Comparison of Published Models
Source: PLoS One. 2017 Jan 13;12(1):e0169437. doi: 10.1371/journal.pone.0169437 (PMC5234801; doi:10.1371/journal.pone.0169437)
Supplement: S1 File — (DOCX) [file pone.0169437.s008.docx]

**S1 File**. The detailed information about the reactions corrected in the pathway-consensus process with glucose as the carbon source.

**Table A.** The direction of reactions changed.

|  | Reaction | Reaction name and direction in initial four models (iJN746, iJP815, iJP962, PpuMBEL1071) | Reaction direction in KEGG, Metacyc and the $\boldsymbol{\Delta}_{\boldsymbol{r}}$G (kJ/ mol) value of reaction | Changed reaction |
| --- | --- | --- | --- | --- |
|  | ATP + Acetate + CoA => AMP + PPI + Acetyl-CoA | R_ACSr (<=>)  --  IR03548 (=>)  GLY_fad (=>) | KEGG (=>)  Metacyc (=>)  -7.4(=>) | R_ACSr (=>)  (iJN746 was changed) |
|  | ATP + Phenylacetic acid + CoA => AMP + PPI + Phenylacetyl-CoA | R_PACCOALr (<=>)  IR01460 (=>)  IR01460 (=>)  R0546 (=>) | KEGG (=>)  Metacyc (=>)  -10.5 (=>) | R_PACCOALr (=>)  (iJN746 was changed) |
|  | Glycine + Tetrahydrofolate + NAD + => 5,10-Methylenetetrahydrofolate + NH_4_ + CO_2_ + NADH + H+ | R_GLYCL (<=>)  RR00772 (<=>)  RR00772 (<=>)  R0190 (<=) | KEGG (=>)  Metacyc (<=>)  -6.6 (=>) | R_GLYCL (=>)  RR00772 (=>)  RR00772 (=>)  (iJN746, iJP815, iJP962 and MBEL1071 were changed) |
|  | Isocitrate + NADP+ => 2-Oxoglutarate + CO_2_ + NADPH + H+ | R_ICDHyr (<=>)  RR00131 (<=>)  RR00131 (<=>)  TCA_icd (<=>) | KEGG (=>)  Metacyc (<=>)  -14.5 (=>) | R_ICDHyr (=>)  RR00131 (=>)  RR00131 (=>)  TCA_icd (=>)  (iJN746, iJP815, iJP962 and MBEL1071 were changed) |
|  | L-Serine => H+ + NH_4_ + Pyruvate | R_ACSr and R_SER_AL (<=>)  IR03538 (=>)  IR03538 (=>)  R0386 (=>) | KEGG (<=>)  Metacyc (=>)  -57.8 (=>) | R_ACSr and R_SER_AL (=>)  (iJN746 was changed) |
|  | 2-Oxoglutarate + Lipoamide => CO_2_ + Succinyldihydrolipoamide | R_AKGDa (<=>)  --  --  -- | KEGG (--)  Metacyc (--)  -32.6 (=>) | R_AKGDa (=>)  (iJN746 was changed) |
|  | O-Acetyl-L-homoserine + Hydrogen sulfide => L-Homocysteine + Acetate | R_METACH (<=>)  IR03622 (=>)  IR03622 (=>)  R0410 (=>) | KEGG (=>)  Metacyc (<=>)  -42.8 (=>) | R_METACH (=>)  (iJN746 was changed) |
|  | Succinyl-CoA + Acetoacetate => Succinate + Acetoacetyl-CoA | R_OCOAT1 (=>)  RR00221 (<=>)  RR00221 (<=>)  R0162 (<=>) | KEGG (<=>)  Metacyc (<=>)  16.3(<=) | R_OCOAT1 (<=>)  (iJN746 was changed) |
|  | Nicotinate + 5-Phospho-alpha-D-ribose 1-diphosphate + ATP + H2O + H+ => Nicotinate D-ribonucleotide + Diphosphate + ADP + Pi | R_NAMNPP (=>)  RR00952 (<=>)  RR00952 (<=>)  R0711 (<=>) | KEGG (<=>)  Metacyc (=>)  -49.0 (=>) | R_NAMNPP (<=>)  (iJN746 was changed) |
|  | ATP + D-Fructose 1-phosphate => ADP + beta-D-Fructose 1,6-bisphosphate | R_FRUK_r (<=>)  IR08910 (=>)  IR08910 (=>)  FRUC_fruK (<=>) | KEGG (=>)  Metacyc (=>)  -17.4 (=>) | R_FRUK_r (=>)  FRUC_fruK (=>)  (iJN746, MBEL1071 were changed) |
|  | 2-Dehydro-3-deoxy-6-phospho-D-gluconate => D-Glyceraldehyde 3-phosphate + Pyruvate | R_EDA (=>)  RR03001 (<=>)  RR03001 (<=>)  ED_eda (<=>) | KEGG (<=>)  Metacyc (=>)  -7.8 (=>) | R_EDA (<=>)  (iJN746 was changed) |
|  | Butanoyl-CoA + FAD => FADH2 + Crotonoyl-CoA | R_ACOAD1f (<=>)  IR00737 (=>)  IR00737 (=>)  -- | KEGG (=>)  Metacyc (<=>)  24.3(<=) | R_ACOAD1f (=>)  (iJN746 was changed) |
|  | Butanoyl-CoA + NAD+ => Crotonoyl-CoA + NADH + H+ | --  RR00733 (<=>)  --  R0158 (<=>) | KEGG (<=)  Metacyc (<=)  62.8 (<=) | RR00733 (<=)  R0158 (<=)  (iJP815 and MBEL1071 were changed) |
|  | 5,10-Methenyltetrahydrofolate + H_2_O => 10-Formyltetrahydrofolate + H+ | R_MTHFC (<=>)  IR00902 (=>)  RR00902 (<=>)  R0754 (<=>) | KEGG (<=>)  Metacyc (<=>)  -6.7(=>) | IR00902 (<=>)  (iJP815 was changed) |
|  | Pyruvate + Hydrogen sulfide + NH_4_ => L-Cysteine + H_2_O | --  IR04850 (=>)  IR04850 (=>)  R0414(<=) | KEGG (<=>)  Metacyc (<=)  39.3 (<=) | IR04850 (<=)  (iJP815 and iJP962 were changed) |
|  | NAD + D-Glyceraldehyde + H_2_O => D-Glycerate + (2.0)H+ + NADH | R_GLYALDDr (<=>)  IR00973 (=>)  IR00973 (=>)  R0228 (<=>) | KEGG (<=>)  Metacyc (<=>)  -41.0 (=>) | IR00973 (<=>)  (iJP815 and iJP962 were changed) |
|  | ATP + Pyruvate + HCO_3_^-^ =>  ADP + PI + Oxaloacetate | R_PC (=>)  RR00181 (<=>)  RR00181 (<=>)  TCA_accC (=>) | KEGG (=>)  Metacyc (=>)  -8.5 (=>) | RR00181 (=>)  (iJP815 and iJP962 were changed) |
|  | 5-Phospho-alpha-D-ribose-1-diphosphate + Uracil => PPI + UMP | R_UPPRT (=>)  RR00617 (<=>)  RR00617 (<=>)  R0334 (=>) | KEGG (<=>)  Metacyc (=>)  -19.9 (=>) | RR00617 (=>)  (iJP815 and iJP962 were changed) |
|  | L-Aspartate + Ammonia => L-Asparagine | R_ASNN (<=)  IR00250 (<=)  IR00250 (<=)  R0196 (<=>) | KEGG (<=)  Metacyc (<=)  29.2(<=) | R0196 (<=)  (PpuMBEL1071  was changed) |
|  | O-Acetyl-L-homoserine + L-Cysteine => L-Cystathionine + Acetate | --  IR01831 (=>)  IR01831 (=>)  R0409 (<=>) | KEGG (=>)  Metacyc (=>)  -41.8 (=>) | R0409 (=>)  (PpuMBEL1071  was changed) |
|  | D-Fructose1-phosphate => Glyceronephosphate + D-Glyceraldehyde | --  RR01486 (<=>)  RR01486 (<=>)  FRUC_fda (=>) | KEGG (<=>)  --  4.1 (=>) | FRUC_fda (<=>)  (PpuMBEL1071  was changed) |
|  | NAD+ + Glycolaldehyde + H_2_O => H+ + NADH + Glycolate | R_GCALDD(=>)  RR00823(<=>)  --  I51(=>) | KEGG (<=)  Metacyc (=>)  -41.5 (=>) | RR00823(=>)  (iJP815 was changed) |
|  | Pyridoxine phosphate + O_2_ => Hydrogen peroxide + Pyridoxal phosphate | R_PDX5Poi (=>)  RR00142 (<=>)  RR00142 (<=>)  R0700 (=>) | KEGG (=>)  Metacyc (=>)  -109.3 (=>) | RR00142 (=>)  RR00142 (=>)  (iJP815 and iJP962 were changed) |
|  | (S)-1-Pyrroline-5-carboxylate + NADH + H+ =>L-Proline + NAD+ | R_P5CR (=>)  RR00795 (<=>)  IR05172 (=>)  R0584 (<=>) | KEGG (<=>)  Metacyc (=>)  -30.4 (=>) | RR00795 (=>)  (iJP815 and PpuMBEL1071 was changed) |

**Table A annotation:**

1. iJN746 had an loop of net production ATP from AMP (Figure 1). The loop was caused by the reactions R_ACSr (ATP + Acetate + CoA => AMP + PPI + Acetyl-CoA), which was reversible in iJN746 but it should be irreversible according to KEGG, Metacyc and the $\Delta_{r}$G value.

5. For the synthesis of L-Serine, iJN746 could generate L-Serine directly from pyruvate so the directions of R_SER_AL and R_SERD_Lr should be changed.

9. Nicotinate is dead-end metabolite in iJN746 because all reactions containing nicotinate were irreversible. According to the other three models we make reaction R_NAMNPP reversible.

11. 2-Dehydro-3-deoxy-phosphogluconate aldolase catalyzes the reversible reaction (2-dehydro -3-deoxy-6-phospho-D-gluconate <=> D-Glyceraldehyde 3-phosphate + Pyruvate) [1, 2] so the reaction R_EDA in iJN746 is changed to be reversible.

12-13. The processes from butanoyl-CoA to crotonoyl-CoA and from crotonoyl-CoA to butanoyl-CoA were [catalyze](javascript:void(0);)d by different enzymes and cofactors. The reactions showed in here should be irreversible so we change the [reversibility](javascript:void(0);) of these two reactions and add them to the models missing them (see Table S6 35 and 36).

15. In iJP962, pyruvate can directly produce cysteine by the reaction IR04850 (Pyruvate + Hydrogen sulfide + NH_4_ => Cysteine + H_2_O) so the direction of this reaction must be wrong.

**Table B.** Reactions and corresponding names and genes were added.

|  | Added reactions | Name of added reactions | Gene of added reactions | Models were added reactions |
| --- | --- | --- | --- | --- |
| 1. 1 | 3-Oxopropionyl-CoA + NADP+ + H_2_O => Malonyl-CoA + NADPH + H+ | ADD_opcoa1 | --(gap filling) | iJN746, iJP815, iJP962 |
|  | 3-Hydroxypropionyl-CoA + NADP+ <=>  3-Oxopropionyl-CoA + NADPH + H+ | ADD_opcoa2 | --(gap filling) | iJN746, iJP815, iJP962 |
|  | 3-Hydroxypropionyl-CoA <=>  Propenoyl-CoA + H_2_O | ADD_opcoa3 | PP_1845 or PP_2136 or PP_2217 or PP_3284 or PP_4030 | iJN746 |
|  | Propanoyl-CoA + FAD + (4.0)H+ =>  FADH_2_ + Propenoyl-CoA | ADD_opcoa4 | PP_2437 or PP_2793 | iJN746 |
|  | NAD+ + Glycolate <=> Glyoxylate + H+ + NADH | ADD_glycolate | PP_0762 | iJN746 |
|  | ATP + Adenylyl sulfate => ADP + 3'-Phosphoadenylyl sulfate | ADD_SO4 | PP_1304 | iJN746 |
|  | D-Fructose 1-phosphate <=> Dihydroxyacetone phosphate + D-Glyceraldehyde | ADD_f1p | PP_4960 | iJN746 |
|  | FAD + 2-Methylpropanoyl-CoA => FADH_2_ + 2-Methylprop-2-enoyl-CoA | ADD_2mp2coa | PP_2437 or PP_2793 | iJN746 |
|  | ATP + Pyridoxine => H+ + Pyridoxine phosphate + ADP | ADD_pydxn1 | PP_5357 | iJN746 |
|  | Pyridoxine + O_2_ => Pyridoxal+H_2_O_2_ | ADD_pydxn2 | PP_1129 | iJN746 |
|  | ATP + Pyridoxal => H+ + Pyridoxal phosphate + ADP | ADD_pydx | PP_5357 | iJN746 |
|  | NAD+ + Glycerol <=> H+ + D-Glyceraldehyde + NADH | ADD_glycerol | PP_5278 or PP_2589 or PP_2680 or PP_5258 or PP_3463 or PP_0545 or PP_2694 or PP_3357 | iJP815 |
|  | H+ + Crotonoyl-CoA + NADH => Butanoyl-CoA + NAD+ | ADD_btcoa | PP_4948 | iJN746, iJP962 |
|  | Butanoyl-CoA + FAD => FADH2 + Crotonoyl-CoA | ADD_btcoa1 | PP_2437 or PP_2793 or PP_4948 | PpuMBEL1071 |
|  | L-Phenylalanine => Phenethylamine + CO_2_ | Add_pac1 | PP_2552 | iJN746 |
|  | Phenethylamine + O_2_ + H_2_O => Phenylacetaldehyde + Ammonia + H_2_O_2_ | Add_pac2 | PP_4983 | iJN746, iJP815, iJP962 |
|  | Phenylacetaldehyde + NAD+ + H_2_O => Phenylacetic acid + NADH + H+ | Add_pac3 | PP_3463 | iJN746, iJP815, iJP962 |
|  | ATP + Acetate + CoA => AMP + PPi + Acetyl-CoA | ADD_acetate1 | PP_2213 or PP_4487 or PP_4702 | iJP815 |
|  | Acetate + ATP => Acetyl phosphate + ADP | ADD_acetate2 | --(gap filling) | iJP815,iJP962,  PpuMBEL1071 |
|  | Folic acid + (2.0)NADPH + (2.0)H+ => Tetrahydrofolate + (2.0)NAD | ADD_folate | PP_5132 | iJN746, iJP815, iJP962 |
|  | Dihydrofolate + NAD+ <=> Folate + NADH + H+ | ADD_folate1 | PP_5132 | iJN746 |
|  | Phosphate + Uridine <=>  alpha-D-Ribose 1-phosphate + Uracil | ADD_uridine | --(Gap filling) | iJP815,iJP962,  PpuMBEL1071 |
|  | alpha-D-Ribose 1-phosphate <=>  D-Ribose 5-phosphate | ADD_uridine1 | PP_1777 or PP_3578 or PP_5288 | iJP962 |
|  | (2.0)Reduced-glutathione + S-Sulfo-L-cysteine <=> L-Cysteine + Oxidized glutathione + H+ + Sulfite | ADD_scys | --(Gap filling) | iJP815,iJP962,  PpuMBEL1071 |
|  | Choline + FAD =>FADH_2_ + Betaine aldehyde | IR09936 | PP_5064 | iJP815 |
|  | NAD+ + Formaldehyde + H_2_O => Formate + (2.0)H+ + NADH | IR00331 | PP_0328 | iJP815 |
|  | Pyruvate + 2-Oxobutanoate + H+ => CO_2_ + S-2-Aceto-2-hydroxybutanoate | IR08875 | PP_4679 and (PP_4680 or PP_1157 or PP_3365 or PP_1394) | iJP815 |
|  | N-Formyl-L-glutamate+H_2_O => Formate + L-Glutamate | IR00279 | PP5029 | iJP962 |
|  | Glycolaldehyde + H_2_O + NAD+ =>  Glycolate + H + NADH | ADD_glycolaldehyde | --(gap filling) | iJP962 |
|  | 5,6-dihydrouracil + NADP+ <=>  H+ + NADPH + Uracil | ADD_uracil1 | PP_4038 | iJP815,iJP962,  PpuMBEL1071 |
|  | 5,6-dihydrouracil + H_2_O => 3-Ureidopropionate + H+ | ADD_uracil2 | --(gap filling) | iJP815,iJP962,  PpuMBEL1071 |
|  | 2-Oxoglutarate + L-Aspartate <=> Oxaloacetate + L-Glutamate | ADD_oaa-asp | --(gap filling) | PpuMBEL1071 |
|  | ATP + L-Glutamine + L-Aspartate => PPI+AMP+ L-Glutamate + L-Asparagine | ADD_asp-asn | PP_1750 | PpuMBEL1071 |
|  | NAD+ + Prephenate => 3-(4-Hydroxyphenyl)pyruvate + NADH + CO_2_ | ADD_tyr1 | PP_1770 | PpuMBEL1071 |
|  | L-Tyrosine + 2-Oxoglutarate <=>  3-(4-Hydroxyphenyl)pyruvate + L-Glutamate | ADD_tyr2 | PP_1972 or PP_3590 or PP_0967 | PpuMBEL1071 |
|  | 3-(4-Hydroxyphenyl)pyruvate + O_2_ => homogentisate +CO_2_ | ADD_tyr3 | PP_2554 or PP_3433 | PpuMBEL1071 |
|  | Hypoxanthine + NAD+ + H_2_O => Xanthine + H+ + NADH | ADD_Hypoxan | PP_4279 and PP_4278 | PpuMBEL1071 |
|  | Formate + ATP + 5-Phosphoribosylglycinamide => Orthophosphate + H+ + ADP + 5-Phosphoribosyl-N-formylglycinamide | ADD_5_phopho | PP_1457 | PpuMBEL1071 |
|  | (2.0)H_2_O_2_ => O_2_ + (2.0)H_2_O | ADD_H2O2 | PP_2887 or PP_0115 or PP_0481 or PP_3668 | PpuMBEL1071 |
|  | Glucose[e] + ATP => Glucose + ADP + Pi | ADD_Glcxt | PP_1018 and (PP_1017 or PP_1016) and PP_1015 | PpuMBEL1071 |
|  | (2.0)Sodium[e] + Sulfate[e] <=> (2.0)Sodium+ Sulfate | ADD_SO4trans | PP_3931 | iJP815,iJP962,  PpuMBEL1071 |
|  | N6-(1,2-Dicarboxyethyl)-AMP <=> AMP + Fumarate | ADD_ASUC1 | PP_4016 | PpuMBEL1071 |
|  | IMP + L-Aspartate + GTP => Orthophosphate + (2.0)H+ + GDP + N6-(1,2-Dicarboxyethyl)-AMP | ADD_ASUC2 | PP_4889 | PpuMBEL1071 |
|  | D-erythro-1-(Imidazol-4-yl)glycerol 3-phosphate => H_2_O + 3-(Imidazol-4-yl)-2-oxopropyl phosphate | ADD_DIMGP1 | PP_0289 | PpuMBEL1071 |
|  | N-(5-Phospho-D-1-ribulosylformimino)-5-amino-1-(5-phospho-D-ribosyl)-4-imidazolecarboxamide + L-Glutamine => H+ + L-Glutamate + 1-(5-Phosphoribosyl)-5  -amino-4-imidazolecarboxamide + D-erythro-1-(Imidazol-4-yl)glycerol 3-phosphate | ADD_DIMGP2 | PP_0290 and PP_0293 | PpuMBEL1071 |
|  | Iron[e] + ATP + H_2_O => Orthophosphate + H+ + ADP + Iron  and Iron[e] => | ADD_heme1 and ADD_heme2 | (PP1078 and (PP4881 or PP5196) and (PP4882 or PP5195)) | iJP815  PpuMBEL1071 |
|  | (2.0)S-Adenosyl-L-methionine + Coproporphyrinogen III => (2.0)5-Deoxyadenosine + Protoporphyrinogen IX + (2.0)L-Methionine + (2.0)H+ + (2.0)CO_2_ | ADD_heme3 | PP4264 or PP5101 | iJP815 |
|  | (2.0)Protoporphyrinogen IX + (3.0)O_2_ => (2.0)Protoporphyrin + (6.0)H_2_O | ADD_heme4 | PP0189 or PP0734 | iJP815  PpuMBEL1071 |
|  | D-Mannose 6-phosphate <=>D-Fructose 6-phosphate | ADD_gdp6dm1 | PP_1277 | iJN746 |
|  | D-Mannose 6-phosphate <=> D-Mannose 1-phosphate | ADD_gdp6dm2 | PP_1777 or PP_5288 | iJN746 |
|  | D-Mannose 1-phosphate + H+ + GDP <=> GDPmannose + Orthophosphate | ADD_gdp6dm3 | PP_1776 or PP_1227 | iJN746 |
|  | GDPmannose => GDP-4-dehydro-6-deoxy-D-mannose + H2O | ADD_gdp6dm4 | PP_1799 | iJN746 |
|  | GDP-6-deoxy-D-mannose + NADP+ <=> H+ + NADPH + GDP-4-dehydro-6-deoxy-D-mannose | ADD_gdp6dm5 | PP_1800 | iJN746, iJP815, PpuMBEL10711071 |
|  | Sedoheptulose 7-phosphate =>D-Glycero-D-manno-heptose 7-phosphate | ADD_adplgmh1 | PP_1323 | iJN746 |
|  | ATP + D-Glycero-D-manno-heptose 7-phosphate =>H+ + ADP + D-Glycero-D-manno-heptose 1,7-bisphosphate | ADD_adplgmh2 | PP_4934 | iJN746 |
|  | D-Glycero-D-manno-heptose 1,7-bisphosphate + H_2_O =>D-Glycero-D-manno-heptose 1-phosphate + Orthophosphate | ADD_adplgmh3 | PP_0059 | iJN746 |
|  | D-Glycero-D-manno-heptose 1-phosphate + ATP + H+ =>Pyrophosphate + ADP-D-glycero-D-manno-heptose | ADD_adplgmh4 | PP_4934 | iJN746 |
|  | ADP-D-glycero-D-manno-heptose =>ADP-L-glycero-D-manno-heptose | ADD_adplgmh5 |  | iJN746 |
|  | D-Arabinose-5-phosphate <=> D-Ribulose-5-phosphate | ADD_cmpmo1 | PP_0957 | iJN746 |
|  | Phosphoenolpyruvate + D-Arabinose-5-phosphate + H_2_O => 3-Deoxy-D-manno-octulosonate-8-phosphate + Orthophosphate | ADD_cmpmo2 | PP_1611 or PP_1807 | iJN746 |
|  | 3-Deoxy-D-manno-octulosonate-8-phosphate + H_2_O => Orthophosphate + 3-Deoxy-D-manno-octulosonate | ADD_cmpmo3 | PP_0956 | iJN746 |
|  | CTP+3-Deoxy-D-manno-octulosonate=>Pyrophosphate+CMP-3-deoxy-D-manno-octulosonate | ADD_cmpmo4 | PP_1902 | iJN746 |
|  | CoA + R-3-Hydroxybutanoyl-ACP <=> ACP + R-3-Hydroxybutanoyl-CoA | ADD_3hbcoa1 | PP_1408 | iJN746 |
|  | (R)-3-Hydroxybutanoyl-ACP => But-2-enoyl-ACP + H_2_O | ADD_3hbcoa2 | PP_4147 or PP_1602 | iJN746 |
|  | UDPglucose <=> UDP-D-galactose | ADD_udpg | PP_3129 | iJN746 |
|  | ATP + H+ + D-Glucose 1-phosphate => Pyrophosphate + ADPglucose | ADD_glycogen1 | --(gap filling) | iJN746 |
|  | ADPglucose => Glycogen + H+ + ADP | ADD_glycogen2 | PP_4050 | iJN746 |
|  | S-Adenosyl-L-methionine+H+ => S-Adenosylmethioninamine + CO_2_ | ADD_spde1 | --(gap filling) | iJN746, iJP962,  PpuMBEL1071 |
|  | S-Adenosylmethioninamine+Putrescine => Spermidine+H++5-Methylthioadenosine | ADD_spde2 | --(gap filling) | PpuMBEL1071 |
|  | 5-Methylthioadenosine + H_2_O => Adenine+5-Methylthio-D-ribose | ADD_spde3 | PP_3254 | iJN746 |
|  | ATP+5-Methylthio-D-ribose => 5-Methylthio-D-ribose-1-phosphate+H++ADP | ADD_spde4 | --(gap filling) | iJN746, PpuMBEL1071 |
|  | 5-Methylthio-D-ribose-1-phosphate <=> 5-Methylthio-5-deoxy-D-ribulose-1-phosphate | ADD_spde5 | PP_1766 | iJN746, PpuMBEL1071 |
|  | 5-Methylthio-5-deoxy-D-ribulose-1-phosphate => 2,3-diketo5-methylthio-1-phosphopentane + H_2_O | ADD_spde6 | --(gap filling) | iJN746, PpuMBEL1071 |
|  | 2,3-diketo5-methylthio-1-phosphopentane + H_2_O => Orthophosphate + (2.0)H+ + 1,2-dihydroxy-3-keto-5-methylthiopentene | ADD_spde7 | --(gap filling) | iJN746, PpuMBEL1071 |
|  | O_2_+1,2-dihydroxy-3-keto-5-methylthiopentene=>Formate+2-keto-4-methylthiobutyrate | ADD_spde8 | PP_1832 | iJN746, PpuMBEL1071 |
|  | 2-keto-4-methylthiobutyrate + L-Glutamate => L-Methionine + 2-Oxoglutarate | ADD_spde9 | PP_3590 or PP_1972 | iJN746, PpuMBEL1071 |

**Table B annotation:**

25-28. In iJP815 and iJP962, 3-Hydroxypropionyl-CoA was dead-end metabolite because it participated in only one reaction to produce Propanoyl-CoA (R0138 3-Hydroxypropionyl-CoA<=> Propenoyl-CoA). There was no reaction [contain](javascript:void(0);)ing 3-Hydroxypropionyl-CoA and Propanoyl-CoA in iJN746. So we add reactions and genes [related](javascript:void(0);) [to](javascript:void(0);) 3-Hydroxypropionyl-CoA and Propanoyl-CoA.

29. Add reaction production glyoxylate from glycolate in iJN746.

30. There are two sulfate-activating enzymes to catalyze the activation of inorganic sulfate to PAPS (adenosine-5-phosphosulfate), namely, ATP-sulfurylase (ATP:sulfate adenylyltransferase; EC 2.7.7.4, sulfate adenylyltransferase) and APS kinase (ATP:adenylylsuifate 3'-phosphotransferase; EC 2.7.1.25, adenylylsulfate kinase) [3] so add the reaction from APS to PAPS and then change the reaction R_APSR (show in 101).

31-35. Add the missing gens and reactions to iJN746.

42-45. In iJP815 and iJP962, acetyl phosphate and folic acid were dead-end metabolites so add the missing reactions 40 and 41. In iJN746 there was no metabolite of folic acid so add 42 and 43.

53. Glycolaldehyde can only be eliminated from the cell in iJN962, but in other three models glycolaldehyde can generate glycolate which is further metabolized. Add the reaction according to other three models.

54-55. 3-Ureidopropionate was dead-end metabolite in iJN746, iJP815 and iJP962. Add reactions production 3-ureidopropionate from uracil.

56. The L-aspartate is synthetized from oxaloacetate [4] but in PpuMBEL1071 the [precursor](javascript:void(0);) of L-aspartate synthesis is acetate (acetate => acetaldehyde => L-threonine => L-cystathionine => O-acetyl-L-homoserine => L-homoserine => 4-phospho-L-aspartate => L-aspartate). After removing the reaction GLY_R2 (Table S8, 91) L-aspartate could not synthetize so we added the reversible reaction 2-Oxoglutarate + L-Aspartate => Oxaloacetate + L-Glutamate to PpuMBEL1071.

58-60. Add the reactions about L-tyrosine synthesis in PpuMBEL1071.

64. In *P. putida* KT2440 glucose transport from the periplasmic space to the cytoplasm via an ABC transport system made up of PP1015, PP1016, PP1017, and PP1108[5,6]. But in PpuMBEL1071 extracellular glucose could directly produce [intracellular](javascript:void(0);) glucose-6-phosphate (TRANS_glk GLCxt + ATP => G6P + ADP). So the glucose ABC transport system should be added.

70. There are no iron transport reactions in iJP815 and PpuMBEL1071.

71. Protoporphyrin is dead-end metabolite in iJP815. It can only be consumed but not be generated.

73-77. GDP-4-dehydro-6-deoxy-D-mannose is dead-end metabolite in iJP815 and PpuMBEL1071. There are no related metabolic pathways about GDP-4-dehydro-6-deoxy-D-mannose in iJN746. Add the corresponding reaction.

78-91. Add the metabolic pathways of ADP-L-glycero-D-manno-heptose, CMP-3-deoxy-D-manno-octulosonate, (R)-3-Hydroxybutanoyl-ACP, UDPglucose and Glycogen to iJN746.

92-100. Spermidine cannot be synthesized in iJN746, PpuMBEL1071 and iJP962 so add corresponding reactions.

**Table C.** Reactions functions were changed.

|  | Name of initial reactions | Initial reactions | After changed reactions |
| --- | --- | --- | --- |
|  | R_APSR in iJN746 | Adenylyl sulfate + Thioredoxin <=> Thioredoxin disulfide + Sulfite + Adenosine 3',5'-bisphosphate | 3'-Phosphoadenylyl sulfate + Thioredoxin <=> Thioredoxin disulfide + Sulfite + Adenosine 3',5'-bisphosphate |
|  | R_PPNCL in iJN746 | D-4-Phosphopantothenate + CTP + L-Cysteine => N-R-4-Phosphopantothenoyl-L-cystein + CDP + H+ + Phosphate | D-4-Phosphopantothenate + CTP + L-Cysteine => N-R-4-Phosphopantothenoyl-L-cystein + CMP + H+ + Diphosphate |
|  | R_PABB in iJN746 | Chorismate + NH_4_ =>  4-amino-4-deoxychorismate + H_2_O | Chorismate + L-Gln =>  4-amino-4-deoxychorismate + L-Glu |
|  | R_MECDPDH in iJN746, IR03226 in iJP815, iJP962 | 2-C-Methyl-D-erythritol-2,4-cyclodiphosphate => 1-Hydroxy-2-methyl-2-butenyl 4-diphosphate + H_2_O | 2-C-Methyl-D-erythritol-2,4-cyclodiphosphate + NADH + H+ => NAD + 1-Hydroxy-2-methyl-2-butenyl 4-diphosphate |
|  | IR02361 in iJP815 | D-4-Phosphopantothenate + L-Cysteine + ATP => Pi + (R)-4-Phosphopantothenoyl-L-cysteine + H+ + ADP | D-4-Phosphopantothenate + L-Cysteine + ATP => PPi + (R)-4-Phosphopantothenoyl-L-cysteine + H+ + AMP |
|  | RR00952 (iJP815 and iJP962), R0711 (MBEL1071) | Pyrophosphate + Nicotinate D-ribonucleotide <=> H+ + Nicotinate + 5-Phospho-alpha-D-ribose 1-diphosphate | Pyrophosphate + Nicotinate D-ribonucleotide + ADP + Orthophosphate <=> H+ + Nicotinate + 5-Phospho-alpha-D-ribose 1-diphosphate + ATP + H_2_O |
|  | R_NADS2,  IR00123, R0717 in iJN746, iJP815,  MBEL1071 | ATP + Deamino-NAD + L-Glutamine + H2O =>AMP + L-Glutamate + H+ + NAD+ + PPI | ATP + Deamino-NAD + NH4 =>AMP+ H+ + NAD+ + PPI |
|  | R_CTPS2 in iJN746 | ATP+ L_Glutamine+ H_2_O + UTP =>  ADP+ CTP+ L_Glutamate+ (2.0)H+ Pi | ATP+Ammonium+UTP =>  ADP + CTP+ H+ Pi |
|  | IR03646 in iJP815, iJP962 | ATP + Carbamate =>  Carbamoyl phosphate + H+ + ADP | ATP + NH4 + CO2 <=> ADP + Carbamoyl phosphate + (2.0)H+ |
|  | IR00314 in iJP815, iJP962 | UTP + ATP + L-Glutamine + H_2_O =>CTP + PI + (2.0)H+ + ADP + L-Glutamate | UTP + ATP + NH4+ =>CTP + PI+H+ + ADP |
|  | R0128 in MBEL1071 | Glycolate + ubiquinone-8 <=> Glyoxylate + ubiquinol-8 | NAD+ + Glycolate <=> Glyoxylate + H+ + NADH |
|  | R0199 in MBEL1071 | SO_4_ + ATP + GTP => Adenylylsulfate+ GDP + PPI + PI | SO_4_ + ATP => Adenylylsulfate + PPI |
|  | GLY_gap in MBEL1071 | D-Glyceraldehyde+PI+NAD+ =>  NADH+3-Phospho-D-glyceroyl | Glyceraldehyde-3-phosphate + NAD+ +PI =>  3-Phospho-D-glyceroyl-phosphate+ NADH |
|  | R0318 in MBEL1071 | (S)-Dihydroorotate+Q=>QH_2_ + Orotate | S)-Dihydroorotate + O_2_ =>H_2_O_2_ + Orotate |
|  | R0249 in MBEL1071 | Aminoimidazole-ribotide + CO_2_+ATP=>  1-(5-Phospho-D-ribosyl)-5-amino-4-imidazolecarboxylate+ADP+Pi | Aminoimidazole-ribotide+CO_2_=>  1-(5-Phospho-D-ribosyl)-5-amino-4-imidazolecarboxylate |
|  | R0749 in MBEL1071 | Folate+NADPH => Tetrahydrofolate + NADP | Folate+(2.0)NADPH => Tetrahydrofolate + (2.0)NADP |
|  | R0500 in MBEL1071 | L-erythro-4-Hydroxyglutamate + NAD+ =>  L-4-Hydroxyglutamate-semialdehyde + NADH | L-erythro-4-Hydroxyglutamate + NADH=>  L-4-Hydroxyglutamate-semialdehyde + NAD+ |
|  | R0132 in MBEL1071 | 3-Oxopropionyl-CoA+O_2_+NADPH <=> Malonyl-CoA + NADP | 3-Oxopropionyl-CoA+O_2_+NADP+ <=> Malonyl-CoA + NADPH |
|  | EXT_gad in MBEL1071 | Gluconate[e] => 2-Dehydro-D-gluconate[e] | Gluconate[e] + FAD => 2-Dehydro-D-gluconate[e] + FADH_2_ |
|  | R0773 in MBEL1071 | Protoporphyrin => Protoheme | Protoporphyrin + Iron => Protoheme |

**Table C annotation:**

101-110. Correct these reactions according to KEGG, Metacyc and Uniprot databases.

111 The corresponding gene and enzyme of this reaction is PP_0762 and 1.1.1.29.In the other three models and in KEGG database the relevant reaction of PP_0762 and 1.1.1.29 is NAD+ + Glycolate <=> Glyoxylate + H+ + NADH.

112-118. Amend the wrong reactions of PpuMBEL1071 according to other three models and databases.

119. D-Gluconate dehydrogenase (EC 1.1.99.3) occurs on the outer surface of cytoplasmic membrane of bacteria and [catalyze](javascript:void(0);)s the reaction from gluconate to 2-Dehydro-D-gluconate (gluconate + acceptor => 2-Dehydro-D-gluconate + reduced acceptor) [7]. The enzyme activity can be also measured with ferricyanide, coenzyme Q (CoQ) or others as an electron acceptor [3] but in PpuMBEL1071 there was no electron acceptor [involved](javascript:void(0);) [in](javascript:void(0);) [the](javascript:void(0);) [reaction](javascript:void(0);). According to other three models we use FADH_2_ as electron acceptor.

**Table D.** Reactions were removed.

|  | Reaction name | Reaction function |
| --- | --- | --- |
|  | R_ALDD2xr, GLY_R2 in iJN746, MBEL1071 | Acetaldehyde + H_2_O + NAD+ <=> Acetate + (2.0)H+ + NADH |
|  | IR00990 in iJP962 | Succinyl-CoA + L-Homoserine => O-Succinyl-L-homoserine + CoA |
|  | IR10058 in iJP962 | O-Succinyl-L-homoserine + Hydrogen-sulfide => Succinate + L-Homocysteine + H+ |
|  | R0575 in MBEL1071 | Prephenate + NAD => Phenylpyruvate + CO_2_ + NADH |
|  | AA_tyrB in MBEL1071 | Phenylpyruvate + L-Glutamate <=>2-Oxoglutarate + L-Tyrosine |
|  | R0258 in MBEL1071 | ATP+Xanthosine 5'-phosphate + NH_3_=> AMP + Pyrophosphate + GMP |

**Table D annotation:**

121. R_ALDD2x and R_ALDD2xr in iJN746 (or R0102 and GLY_R2 in PpuMBEL1071) which correspond to the same genes and enzymes and the only difference between them was that R_ALDD2x and R0102 were irreversible but R_ALDD2xr and GLY_R2 was [reversible](javascript:void(0);). According to Metacyc and the $\Delta$rG value (-50.4 kJ/ mol) of this reaction we remove the reaction R_ALDD2xr in iJN746 and GLY_R2 in PpuMBEL1071.

122-123. In iJP962, there were two pathways from L-homoserine to L-homocysteine. The one pathway was L-homoserine => O-Succinyl-L-homoserine => L-Homocysteine and the other pathway was L-homoserine => O-Acetyl-L-homoserine => L-Homocysteine. In *P.putida* only contain the coding gene (PP5097) from L-homoserine to O-Acetyl-L-homoserine but do not contain the coding gene from L-homoserine to O-Succinyl-L-homoserine. So removed IR00990, IR10058.

124. There are two similar reactions from prephenate to phenylpyruvate R0575 (Prephenate + NAD => NAD Phenylpyruvate + CO_2_ + NADH) and AA_tyrA (Prephenate => CO_2_ + Phenylpyruvate). According to other three models and the KEGG database the reaction AA_tyrA is right.

125. In *P. putida* L-tyrosine was synthesized from prephenate through two steps (ADD_tyr1 and ADD_tyr2) but in PpuMBEL1071 only one reaction AA_tyrB (Phenylpyruvate + GLU => 2-Oxoglutarate + L-Tyrosine) was needed to implement this process. After adding reactions ADD_tyr1 and ADD_tyr2 we removed reaction AA_tyrB.

126. In PpuMBEL10711071there were two reactions R0258 (ATP+Xanthosine 5'-phosphate + NH3 => AMP + Pyrophosphate + GMP) and R0257 (Xanthosine5'-phosphate + ATP + GLN => GMP + GLU + AMP + Pyrophosphate) from Xanthosine 5'-phosphate to GMP but in other three models there was only ome reaction the same as R0257. So we remove the reaction R0258.

**References**

[1] Meloche H P. Bromopyruvate Inactivation of 2-Keto-3-deoxy-6-phosphogluconic Aldolase. I. Kinetic Evidence for Active Site Specificity*[J]. Biochemistry, 1967, 6(8): 2273-2280.

[2] Meloche H P, Wood W A. Crystallization and characteristics of 2-keto-3-deoxy-6-phosphogluconic aldolase[J]. Journal of Biological Chemistry, 1964, 239(10): 3515-3518.

[3] Segel I H, Renosto F, Seubert P A. Sulfate-activating enzymes[J]. Methods in enzymology, 1987, 143: 334-349.

[4] Appels M A, Haaker H. Glutamate Oxaloacetate Transaminase in Pea Root Nodules Participation in a Malate/Aspartate Shuttle between Plant and Bacteroid[J]. Plant physiology, 1991, 95(3): 740-747.

[5] del Castillo T, Ramos J L, Rodríguez-Herva J J, et al. Convergent peripheral pathways catalyze initial glucose catabolism in Pseudomonas putida: genomic and flux analysis[J]. Journal of bacteriology, 2007, 189(14): 5142-5152.

[6] Dos Santos V A P, Heim S, Moore E R B, et al. Insights into the genomic basis of niche specificity of Pseudomonas putida KT2440[J]. Environmental microbiology, 2004, 6(12): 1264-1286.

[7] Matsushita K, Shinagawa E, Ameyama M. D-Gluconate dehydrogenase from bacteria, 2-keto-D-gluconate-yielding, membrane-bound[J]. Methods in enzymology, 1982, 89: 187.
