# Supplementary material for: Pathway-Consensus Approach to Metabolic Network Reconstruction for Pseudomonas putida KT2440 by Systematic Comparison of Published Models
Source: PLoS One. 2017 Jan 13;12(1):e0169437. doi: 10.1371/journal.pone.0169437 (PMC5234801; doi:10.1371/journal.pone.0169437)
Supplement: S2 File — (DOCX) [file pone.0169437.s009.docx]

**S2 File.** The detailed information about reactions corrected when 48 substrates were used as individual carbon sources.

**Table A.** The reactions with direction changes.

|  | **Reaction** | **Reaction name and direction in initial four models (iJN746, iJP815, iJP962, PpuMBEL1071)** | **Reaction direction in KEGG, Metacyc and the** $\boldsymbol{\Delta}_{\boldsymbol{r}}$**G (kJ/ mol) value of reaction** | **Changed reaction** |
| --- | --- | --- | --- | --- |
|  | 6-Phospho-D-gluconate => 2-Dehydro-3-deoxy-6-phospho-D-gluconate + H_2_O | R_EDD (=>)  IR01115 (=>)  IR01115 (=>)  ED_edd (<=>) | KEGG (=>)  Metacyc (=>)  -38.3(=>) | ED_edd (=>) (PpuMBEL1071 was changed) |
|  | 10-Formyltetrahydrofolate + 5'-Phosphoribosylglycinamide => Tetrahydrofolate + 5'-Phosphoribosyl-N-formylglycinamide | R_GARFT (<=>)  RR02406 (<=>)  RR02406 (<=>)  R0246 (=>) | KEGG (<=>)  Metacyc (<=>)  -15.9(=>) | R0246 (<=>)  (PpuMBEL1071 was changed) |
|  | ATP + L-Citrulline + L-Aspartate => AMP + PPi + N-(L-Arginino)succinate | R_ARGSS (=>)  IR03797 (=>)  IR03797 (=>)  R0592 (<=>) | KEGG (=>)  Metacyc (=>)  0.9(<=>) | R0592 (=>)  (PpuMBEL1071 was changed) |
|  | ATP + Acetyl-CoA + HCO_3_^-^ <=> ADP + Pi + Malonyl-CoA | R_ACCOACr (<=>)  RR00438 (<=>)  RR00438 (<=>)  R0203 (=>) | KEGG (=>)  Metacyc (=>)  -6.0 (=>) | R_ACCOACr (=>)  RR00438 (=>)  RR00438 (=>)  (iJN746, iJP815 and iJP962 were changed) |
|  | Chorismate => 4-Hydroxybenzoate + Pyruvate | --  RR03636 (<=>)  RR03636 (<=>)  R0797 (=>) | KEGG (<=>)  Metacyc (=>)  -193.4(=>) | RR03636 (=>)  (iJP815 and iJP962 were changed) |

**Table A annotation:**

1-5. The reaction direction in KEGG, Metacyc database and $\Delta$ $\Delta$rG value were used as a reference to decide the direction of reactions.

**Table B.** Reactions and corresponding names and genes were added.

|  | Added reactions | Name | Gene | Models were added reactions |
| --- | --- | --- | --- | --- |
|  | NAD+ + Formaldehyde + H_2_O => Formate + (2.0)H+ + NADH | ADD_Choline | PP_0328 | iJN746 |
|  | L-Alanine[p] <=> L-Alanine[c] | ADD_Ala | --(gap filling) | iJN746 |
|  | NAD + Quinate => 3-Dehydroquinate + NADH + H+ | ADD_Quinate | PP_3569 | iJN746 |
|  | Chorismate => 4-Hydroxybenzoate + Pyruvate | ADD_Hydroxybenzoate | PP_5317 | iJN746 |
|  | Putrescine + 2-Oxoglutarate => 4-Aminobutanal + L-Glutamate | ADD_4abut1 | PP_4223 | iJN746, MBEL1071 |
|  | 4-Aminobutanal + NAD+ => 4-Aminobutanoate + NADH + H+ | ADD_4abut2 | PP_5278 or PP_2589 or PP_2680 or PP_5258 or PP_3463 or PP_0545 or PP_2694 or PP_3357 | iJN746 |
|  | L-Ornithine => Putrescine + CO_2_ | ADD_4abut3 | PP_0864 | MBEL1071 |
|  | 2-Hydroxy-3-oxopropanoate + NADH => D-Glycerate + NAD+ | ADD_2-hy-3 | PP_4299 | iJP815, iJP962 |
|  | Gallate + O_2_ => 4-Oxalomesaconate | ADD_gallate1 | PP_2518 | MBEL1071 |
|  | 4-Oxalomesaconate => 2-Hydroxy-4-carboxyhexa-2,4-dienedioate | ADD_gallate2 | PP_2513 | MBEL1071 |
|  | H_2_O + 2-Hydroxy-4-carboxyhexa-2,4-dienedioate => 4-Carboxy-4-hydroxy-2-oxoadipate | ADD_gallate3 | PP_2515 | iJP815, iJP962, MBEL1071 |
|  | 4-Carboxy-4-hydroxy-2-oxoadipate => oxaloacetate + pyruvate | ADD_gallate4 | PP_2514 | iJP815, iJP962, MBEL1071 |
|  | H+ + NADH + O_2_ + Toluene => Benzyl alcohol + H_2_O + NAD | ADD_Toluene1 | -- | iJP815, iJP962, MBEL1071 |
|  | Benzyl alcohol + NAD => Benzaldehyde + H+ + NADH | ADD_Toluene2 | -- | iJP815, iJP962, MBEL1071 |
|  | Benzaldehyde + H_2_O + NAD => Benzoate + (2.0)H+ + NADH | ADD_Toluene3 | -- | iJP815, iJP962, MBEL1071 |
|  | Betaine + L-Homocysteine => N-N-Dimethylglycine + L-Methionine | ADD_Choline1 | -- | iJP815, iJP962, MBEL1071 |
|  | N-N-Dimethylglycine + FAD + H_2_O <=> FADH2 + Formaldehyde + Sarcosine | ADD_Choline2 | -- | iJP815, iJP962, MBEL1071 |
|  | H_2_O + L_Methionine => 2-Oxobutanoate + Methanethiol + Ammonium | ADD_Choline3 | PP_1308 | iJP815, iJP962, MBEL1071 |
|  | Methanethiol + (3.0)H_2_O + (3.0)NADP+ <=> (4.0)H + Methanesulfonate+ (3.0)NADPH | ADD_Choline4 | -- | iJP815, iJP962, MBEL1071 |
|  | H + NADPH + Methanesulfonate + O_2_ => Formaldehyde + NADP+ + H_2_O + Sulfite | ADD_Choline5 | PP_0238 | iJP815, iJP962, MBEL1071 |
|  | H_2_O + O_2_ + Sarcosine => Formaldehyde+Glycine+H_2_O_2_ | ADD_Choline6 | PP_0323 and PP_0324 and PP_0325 and PP_0326 and PP_3775 | MBEL1071 |
|  | Phenylacetyl-CoA + O_2_ + NADPH + H+ =>2-(1,2-Epoxy-1,2-dihydrophenyl)acetyl-CoA + H2O + NADP+ | ADD_Phenylacetate1 | PP_3274 and PP_3275 and PP_3276 and PP_3277 and PP_3278 | iJP815, iJP962, MBEL1071 |
|  | 2-(1,2-Epoxy-1,2-dihydrophenyl)acetyl-CoA + (3.0)H_2_O + NADP+ + CoA+ => NADPH + H+ + Acetyl-CoA + (3S)-3-Hydroxyadipyl-CoA | ADD_Phenylacetate2 | PP_3283 and PP_3726 and PP_3270 and PP_1845 and PP_2217 and PP_3284 and PP_4030 | iJP815, iJP962, MBEL1071 |
|  | (3S)-3-Hydroxyadipyl-CoA + NAD+ => 3-Oxoadipyl-CoA + NADH + H+ | ADD_Phenylacetate3 | PP_3282 and PP_3755 | iJP815, iJP962, MBEL1071 |
|  | ATP+ COA+ Hexadecanoate => AMP + Palmitoyl-CoA + Pi | ADD_Hexa1 | ( PP_2213 or PP_4549 ) or PP_4550 | iJP815, iJP962 |
|  | H_2_O + NAD+ + Vanillin => (2.0)H+ + NADH + Vanillate | ADD_Conif1 | PP_3357 | iJP815, iJP962 |
|  | Feruloyl-CoA + H_2_O =>Acetyl-CoA + Vanillin | ADD_Conif2 | PP_3358 | iJP815, iJP962 |
|  | ATP + COA + Ferulate => AMP + Feruloyl-CoA + Diphosphate | ADD_Conif3 | PP_3356 | iJP815, iJP962 |
|  | Coniferyl aldehyde + H_2_O + NAD+ => Ferulate+ (2.0)H+ + NADH | ADD_Conif4 | PP_5120 | iJP815, iJP962 |
|  | Coniferol + NAD+ => Coniferyl aldehyde + H+ + NADH | ADD_Conif5 | -- | iJP815, iJP962 |

**Table B annotation:**

6. In iJN746 formaldehyde cannot consume and directly discharge but other three models can consume formaldehyde by the reaction NAD+ + Formaldehyde + H_2_O => Formate + (2.0)H+ + NADH. Add this reaction and corresponding gene PP0328 to iJN746.

7. iJN746 cannot use L-alanine as [substrate](javascript:void(0);) because L-alanine in [periplasm](javascript:void(0);) could not be transported to [cytoplasm](javascript:void(0);). After adding the transport reaction L-alanine[p] <=> L-alanine[c] iJN746 could utilize L-alanine as [substrate](javascript:void(0);).

8. Add the quinate consuming reaction NAD + Quinate => 3-Dehydroquinate + NADH + H+ which exists in other three models.

10-12. Add missing 4-Aminobutanoate metabolic reactions and genes in iJN746.

14-20. iJP815, iJP962 and PpuMBEL1071 cannot in silico grow on gallate and toluene so add corresponding reactions in the three models.

21-26. iJP815, iJP962 and PpuMBEL1071 cannot in silico grow on choline because choline can [produce](javascript:void(0);) betaine which become dead-end metabolite. Add reactions [related](javascript:void(0);) [to](javascript:void(0);) betaine consumption.

27-29. iJP815, iJP962 and PpuMBEL1071 cannot in silico grow on phenylacetate because phenylacetate can [produce](javascript:void(0);) phenylacetyl-CoA which become dead-end metabolite. So the reactions [related](javascript:void(0);) [to](javascript:void(0);) phenylacetyl-CoA consumption were added.

30-35. iJP815, iJP962 cannot in silico grow on hexadecanoate, vanillin and coniferyl alcohol. So the reactions [related](javascript:void(0);) [to](javascript:void(0);) them were added.

**Table C.** Reactions functions were changed.

|  | Name of initial reaction | Initial reaction | Changed reaction |
| --- | --- | --- | --- |
|  | R_PHACOAOR in iJN746 | Phenylacetyl-CoA + O_2_ + H_2_ => 2-(1,2-Epoxy-1,2-dihydrophenyl)acetyl-CoA | Phenylacetyl-CoA + O_2_ + NADPH + H+ => 2-(1,2-Epoxy-1,2-dihydrophenyl)acetyl-CoA + H2O + NADP+ |
|  | R_DHPACCOAHIT in iJN746 | 2-(1,2-Epoxy-1,2-dihydrophenyl)acetyl-CoA + (2.0)H_2_O + CoA+ => H_2_ + H+ + Acetyl-CoA + (3S)-3-Hydroxyadipyl-CoA | 2-(1,2-Epoxy-1,2-dihydrophenyl)acetyl-CoA + (3.0)H2O + NADP+ + CoA+ => NADPH + H+ + Acetyl-CoA + (3S)-3-Hydroxyadipyl-CoA |
|  | R_HADPCOADH in iJN746 | (3S)-3-Hydroxyadipyl-CoA => 3-Oxoadipyl-CoA + H_2_ | (3S)-3-Hydroxyadipyl-CoA + NAD+ => 3-Oxoadipyl-CoA + NADH + H+ |

**Table C annotation:**

36-38. For the three reactions in iJN746, replace H_2_ with NADH(NADPH).

**Table D.** Reactions were removed.

|  | Reaction name | Reaction function |
| --- | --- | --- |
|  | R_OBDHc in iJN746 | 2-Oxobutanoate + COA + NAD+ => CO_2_ + NADH + Propanoyl-CoA |
|  | R_CAT23DOX in iJN746 | Catechol + O_2_ => 2-Hydroxymuconate semialdehyde |

**Table D annotation:**

39-40. The corresponding genes and enzyme of the two reactions are not annotated in *P. putida* and they do not exist in iJP815, iJP962 and PpuMBEL1071 so remove them from iJN746.
